# Molecular characterization of a bipartite double-stranded RNA virus and its satellite-like RNA co-infecting the phytopathogenic fungus *Sclerotinia sclerotiorum*

**DOI:** 10.3389/fmicb.2015.00406

**Published:** 2015-05-06

**Authors:** Lijiang Liu, Qihua Wang, Jiasen Cheng, Yanping Fu, Daohong Jiang, Jiatao Xie

**Affiliations:** State Key Laboratory of Agricultural Microbiology and The Provincial Key Lab of Plant Pathology of Hubei Province, College of Plant Science and Technology, Huazhong Agricultural UniversityWuhan, China

**Keywords:** *Sclerotinia sclerotiorum*, dsRNA mycovirus, botybirnavirus, *Botrytis porri*, RNA virus 1, satellite-like RNA

## Abstract

A variety of mycoviruses have been found in *Sclerotinia sclerotiorum*. In this study, we report a novel mycovirus *S. sclerotiorum* botybirnavirus 1 (SsBRV1) that was originally isolated from the hypovirulent strain SCH941 of *S. sclerotiorum*. SsBRV1 has rigid spherical virions that are ∼38 nm in diameter, and three double-stranded RNA (dsRNA) segments (dsRNA1, 2, and 3 with lengths of 6.4, 6.0, and 1.7 kbp, respectively) were packaged in the virions. dsRNA1 encodes a cap-pol fusion protein, and dsRNA2 encodes a polyprotein with unknown functions but contributes to the formation of virus particles. The dsRNA3 is dispensable and may be a satellite-like RNA of SsBRV1. Although phylogenetic analysis of the RdRp domain demonstrated that SsBRV1 is related to *Botrytis porri* RNA virus 1 (BpRV1) and Ustilago maydis dsRNA virus-H1, the structure proteins of SsBRV1 do not have any significant sequence similarities with other known viral proteins with the exception of those of BpRV1. SsBRV1 carrying dsRNA3 seems to have no obvious effects on the colony morphology, but can significantly reduce the growth rate and virulence of *S. sclerotiorum*. These findings provide new insights into the virus taxonomy, virus evolution and the interactions between SsBRV1 and the fungal hosts.

## Introduction

Mycoviruses (or fungal viruses) are widely distributed in fungal groups and typically contain RNA genomes. Mycoviruses with RNA genome includes positive single-stranded RNA (+ssRNA) virus and double-stranded RNA (dsRNA) virus. Meantime, an increasing number of novel mycoviruses have been reported and showed some unique molecular and biological attributes as well as taxonomic considerations (Chiba et al., 2009; Yu et al., 2010; Lin et al., 2012; Wu et al., 2012; Hu et al., 2014; Liu et al., 2014; Zhang et al., 2014a,b). Mycoviruses with a DNA genome and a negative single-stranded RNA (-ssRNA) genome were also recently reported in the filamentous fungi *Sclerotinia sclerotiorum* (Yu et al., 2013; Liu et al., 2014). Mycoviruses with dsRNA viruses presently comprise six families, i.e., *Totiviridae*, *Partitiviridae*, *Megabirnaviridae*, *Chrysoviridae*, *Quadriviridae,* and *Reoviridae* (Ghabrial et al., 2015). A proposed dsRNA virus family Botybirnaviridae has been presented for encompassing the newly reported bipartite dsRNA virus *Botrytis porri* botybirnavirus 1 (BpRV1; Wu et al., 2012). Botybirnaviridae presently includes only one member, i.e., BpRV1, which showed some unique molecular and biological properties different from those of all known dsRNA viruses.

In general, mycoviruses with dsRNA genome do not cause any obvious symptoms to their hosts (cryptic infection or latent infection; Nuss, 2005; Ghabrial and Suzuki, 2009). However, dsRNA viruses, such as mycoreovirus 1 (MyRV1) and mycorevirus 3 (MyRV3), *R. necatrix* megabirnavirus 1 (RnMBV1), *Magnaporthe oryzae* chrysovirus 1 (MoCV1), *B. porri* RNA virus 1 (BpRV1) and *S. sclerotiorum* partitivirus 1 (SsPV1), lead to seriously debilitating symptoms in their fungal hosts and show great potential to control the fungal diseases (Osaki et al., 2002; Hillman et al., 2004; Nuss, 2005; Chiba et al., 2009; Urayama et al., 2010; Wu et al., 2012; Xiao et al., 2014). With the increasing number of novel mycoviruses reported, dsRNA mycoviruses seem to be divergent in biological properties and molecular characteristics.

*Sclerotinia sclerotiorum* is a phytopathogenic fungus that has a wide host range covering 64 genera of plants and more than 450 species (Bolton et al., 2006). *Sclerotinia* diseases has given rise to a huge economic cost every year, and are still difficult to be controlled efficiently due to the lack of resistant cultivars and the environmental threat resulting from the abuse of fungicides. Several viruses have been isolated from *S. sclerotiorum* and characterized at the molecular and biological level, e.g., members of the families *Partitiviridae* (Liu et al., 2012; Xiao et al., 2014), *Hypoviridae* (Xie et al., 2011; Khalifa and Pearson, 2014; Marzano et al., 2015), *Narnaviridae* (Xie and Ghabrial, 2012; Khalifa and Pearson, 2013; Xu et al., 2015), and *Alphaflexiviridae* (Xie et al., 2006), and some unassigned viruses (Liu et al., 2009, 2014; Yu et al., 2010; Hu et al., 2014). These findings indicate the large number and diversity of mycoviruses in *S. sclerotiorum* and provide novel insights into the diversity and evolution of viruses as well as the interaction between mycoviruses and their fungal hosts (Jiang et al., 2013).

*Sclerotinia sclerotiorum* strain SCH941, which was isolated from a sclerotium on diseased rapeseed from Sichuan Province, exhibits a debilitated phenotype with lower growth rate, an abnormal colony and few sclerotia formation. Multiple dsRNA segments were detected in the mycelia of strain SCH941 with sizes ranging from 6.5 to 1.2 kbp. Sequence cloning and analysis showed that strain SCH941 is simultaneously infected by two phylogenetically unrelated mycoviruses, namely a bipartite dsRNA virus and a reovirus. In this study, we determined the complete sequence of the bipartite dsRNA virus and analyzed its genome organization, virion morphology, phylogeny, and biological effect on the host.

## Materials and Methods

### Fungal Strains

*Sclerotinia sclerotiorum* strain SCH941 was isolated from a sclerotium collected from a diseased rapeseed (*Brassica napus*) plant in Yingjing County, Sichuan Province, PR China. Strains SCH941R6 and SCH941R7 were derived from the protoplast regeneration of strain SCH941. Strain Ep-1PNA367, a virus-free strain and a single-ascospore isolate derived from Ep-1PN (Li et al., 1996), shows normal colony formation and strong virulence. All *S. sclerotiorum* isolates were cultured on potato dextrose agar (PDA) at 20–22∘C and stored on PDA slants at 4∘C.

### Biological Charateristics and Virulence Assay

The growth rate and virulence test of different strains were measured as the method described by Xiao et al. (2014). More than three replicates were conducted for each treatment. To assess the colony morphology, freshly grown mycelial agar plugs were transferred onto the fresh PDA medium, and cultured on the same conditions (20–22∘C) for 10 days.

### dsRNA Isolation, Molecular Cloning, Sequencing Analysis

Double-stranded RNA isolation, purification, cDNA cloning, and sequencing were performed as previously described by Xie et al. (2011). The terminal sequence was determined following the method described by Potgieter et al. (2009) with minor modifications. Then, 200–500 ng of dsRNA purified from strain SCH941R6 was ligated to 30 pmol of the oligonucleotide primer PC3-T7 loop (5′-p-GGATCCCGGGAATTCGGTAATACGACTCACTATATTTTTATAGTGAGTCGTATTA -OH-3′) in a reaction mixture containing 50 mM Tris-HCl (pH 7.5), 10 mM MgCl_2_, 10 mM DTT, 1 mM ATP, 20U RNase inhibitors, 25% PEG4000 (W/V), and 40 U T4 RNA ligase (TaKaRa, China) and incubated at 4–8∘C for 18 h. The reaction mixture was then supplemented with 600 μl of DEPC-treated double distilled water and extracted using an equal amount of chloroform. The supernatant was collected, supplemented with an equal amount of isopropanol and 0.1 amount (V/V) of 3 M NaAc solution (pH 5.2), and precipitated at -20∘C for 30 min. After centrifugation, the precipitates were collected, washed with 70% ethanol (V/V) and re-dissolved in 16 μl of DEPC-treated double distilled water. The first cDNA synthesis was conducted in a reaction mixture containing 200 U RevertAid^TM^ Reverse Transcriptase (Fermentas, USA), 50 mM Tris-HCl (pH8.0), 50 mM KCl, 4 mM MgCl_2_, 10 mM DTT, 1 mM dNTPs, 20 U RiboLock RNase Inhibitor^TM^ (Fermentas, USA), and distilled water in a final volume of 25 μl. The mixture was incubated at 42∘C for 1 h, 50∘C for 10 min, and 65∘C for 10 min. The cDNA was amplified using the PC2 primer (5′-p-CCGAATTCCCGGGATCC-3′) complementary to the oligonucleotide PC3-T7 loop and a sequence-specific primer corresponding to the 5′-and 3′-terminal sequences of the dsRNA. Sequencing was performed through the dideoxynucleotide termination method using a Big Dye Terminator Sequencing kit (BigDye terminator V. 2.0; ABI) and an ABI PRISM377-96 automated sequencer (BGI).

### Sequence Analysis and Phylogenetic Analysis

Sequence assembly, ORF identification, and secondary structure analysis were performed using DNAMAN and RNA Structure software (version 4.6; Mathews et al., 2004). Sequence similarity analysis was conducted using the online BLAST program^1^. Multiple sequence alignments were performed in the M-Coffee server^2^ and modified using the Clustal_X software by manually removing any gaps. The best-fit model (LG+I+G+F) of protein evolution was obtained using Akaike’s information criterion (AIC) and searched using the ProtTest server^3^ (Abascal et al., 2005). The phylogenetic tree was created in PhyML 3.0 using the maximum likelihood method and the appropriate substitution mode (Guindon et al., 2010). The phylogenetic tree was visualized and refined with MEGA 5.0 (Tamura et al., 2011).

### Purification of Virus Particles and Characterization of Coat Protein

The isolation and purification of viral particles were performed following the method described by Yu et al. (2010) with minor modification. Strain SCH941R6 was grown in PDB or on sterilized cellophane placed on PDA for 7–10 days. The virus stocks were further purified using a sucrose concentration gradient ranging from 20 to 40% (W/V) at 50,000*g*, 4∘C for 2 h. Each gradient was individually collected, extracted using phenol and chloroform and subjected to agarose gel-electrophoresed analysis to detect viral dsRNA. The gradient containing viral particles was diluted with sodium phosphate buffer (0.1 M, pH 7.0) and re-centrifuged at 210,000*g* and 4∘C for 1.5 h. The fractions containing virus particles were carefully collected and re-suspended using 200 μl sodium phosphate buffer (0.1 M, pH 7.0). The virus particles were stained with 2% (W/V) phosphotungstic acid (PTA) and observed using a transmission electron microscope (Model Tecnai G2 20; FEI Company). To identify the structural proteins of SsBRV1 virions, viral particles were purified from SsBRV1-infected strain Ep-1PNA367 carrying satellite RNA and virus-free Ep-1PNA367 was used as the negative control following the same method. polypeptide mass fingerprint-mass spectrum (PMF-MS) analysis of the structural proteins was performed following a previously described method (Liu et al., 2014).

### Protoplast Preparation and Transfection

The virus-free strain Ep-1PNA367 was used as the recipient in this experiment. Protoplasts preparation and transfection using purified viral particles were performed according to a previously described method (Yu et al., 2010). The specific primers BRV1S1-F (5′-CCAGGGCAGTTGCTACAGTCAT-3′) and BRV1S1-R (5′-TTGCCGCAGAGGGGAATC-3′) were used for dsRNA1 detection, the specific primers BRV1S2-F (5′-TCCCCGAGCAACTCAAAAACC-3′) and BRV1S2-R (5′-GCACGATTTAGTGCTGCGGTTT-3′) were used for dsRNA2 detection, and the specific primers BRV1S3-F (5′-TTATCACTCGCACTTCACACGCAG-3′), and BRV1S3-R (5′-GCCCTCAACGCCTTCGTAAAAG-3′) were used for dsRNA3 detection.

### Northern Blot Analysis

For Northern hybridization analysis, dsRNAs were separated on a 0.7% agarose gel and run in 0.5x TBE buffer for 16 h at 50 V. The gel was then denatured in 0.1 M NaOH for 30 min and neutralized in 0.1 M Tris-HCl (pH 8.0) for 30 min. The RNAs were transferred to an Amersham Hybond-N+nylon membrane (GE Healthcare) by capillary action. Digoxigenin (Dig)-labeled DNA preparation, prehybridization, and hybridization were performed as the user manuual (GE Healthcare). The probe-RNA hybrids were detected using a CDP-Star kit (GE Healthcare). Probe 1 was amplified using the specific primers BRV1S1-F (5′-CCAGGGCAGTTGCTACAGTCAT-3′) and BRV1S1-R (5′-TTGCCGCAGAGGGGAATC-3′), probe 2 was amplified using the specific primers BRV1S2-F (5′-TCCCCGAGCAACTCAAAAACC-3′), and BRV1S2-R (3′-GCACGATTTAGTGCTGCGGTTT-3′), and probe 3 was amplified using the primers BRV1-p1-F (5′-GCAATAAAAAGCACAGCCGG-3′) and BRV1-p1-R (5′-TGTTGTGTTATTTGGTATGTTGATCG-3′).

## Results

### dsRNAs in *S. sclerotiorum* Strain SCH941

**FIGURE 1:**
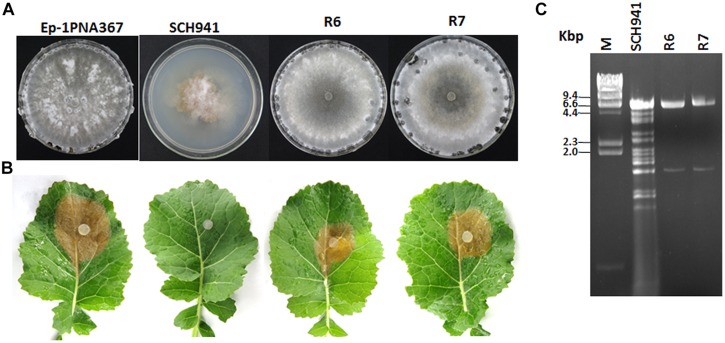
**Colony morphology, virulence, and virus content of the SCH941 strains and the derived isolates. (A)** Colony morphology. The colony morphology of virus-free strain Ep-1PNA367 was used as a positive control. All of the strains were cultured on potato dextrose agar (PDA) at 20–22∘C for 10 days. **(B)** Virulence test on the detached leaves of rapeseed. Strain Ep-1PNA367 was used as a positive control. The photographs were taken after inoculation at 20–22∘C for 3 days. **(C)** Agarose gel-electrophoresed analysis of dsRNA profiles isolated from strains SCH941 and SCH941R6 and SCH941R7.

**FIGURE 2:**
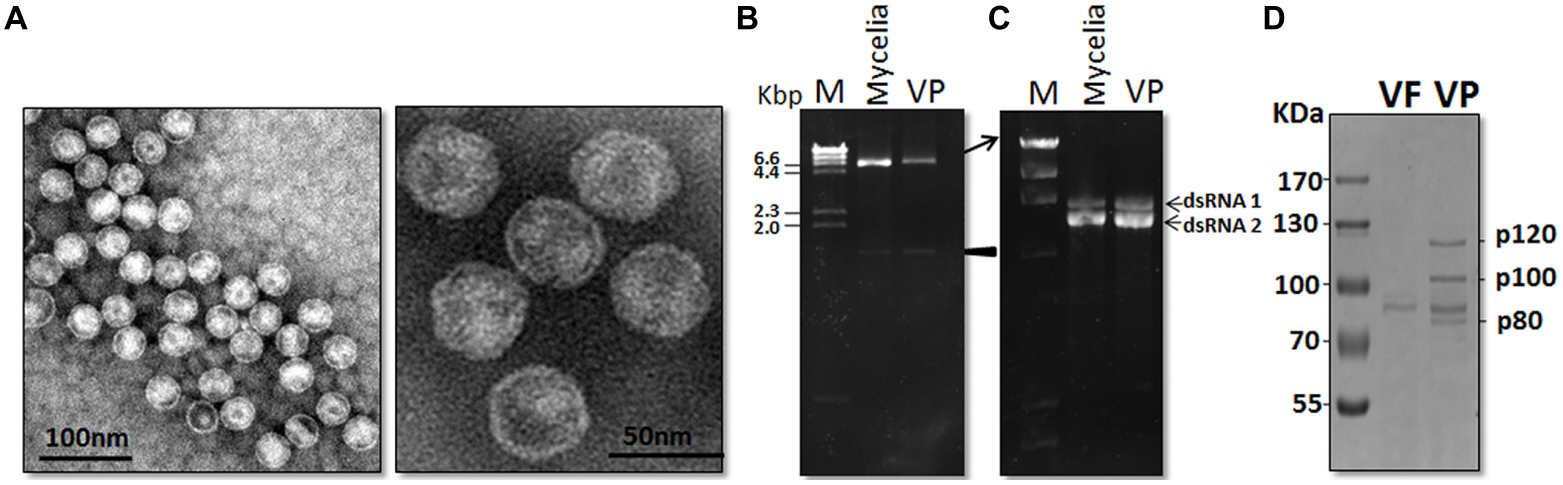
**Composition of virus particles of SsBRV1. (A)** TEM observation of virus particles. **(B)** 1% agarose gel-electrophoresed analysis of dsRNA profiles from mycelia and viral particles. Hind III-digested DNA was used as the molecular marker. **(C)** 0.7% agarose gel-electrophoresed analysis of the upper dsRNA band run in 0.5x TBE buffer for 16 h at 50 V. **(D)** 10% SDS-PAGE gel-electrophoresed analysis of the protein components of viral particles purified from SsBRV1-infected transfectant (lane VP). Fractions followed the same method as virus particles purification from the isogenic strain Ep-1PNA367 was also analyzed (lane VF). The molecular weight of the protein bands was estimated by the protein markers.

Strain SCH941 exhibits an abnormal morphology and is incapable of causing lesions on detached leaves of rapeseed (**Figures 1A,B**). Multiple dsRNA segments with sizes ranging from 6.6 to 1.6 kbp were detected from strain SCH941. To examine whether these dsRNA segments are responsible for the hypovirulence of strain SCH941, a protoplast regeneration technique was introduced. The results showed that some protoplast regenerants were cured, and exhibited a normal phenotype and strong virulence on detached leaves of rapeseed (**Figures 1A,B**). dsRNA isolation revealed that two dsRNA segments with sizes of 6.6 and 1.6 kbp were extracted from those cured regenerants (such as SCH941R6 and R7), whereas the other dsRNA segments were eliminated (**Figure 1C**). Thus, 6.6- and 1.6 kbp-dsRNA segments may not be the hypovirulence factors of strain SCH941. Agarose gel-electrophoresed analysis further revealed that the 6.6 kbp-dsRNA bands were actually composed of two dsRNA segments (temporarily named dsRNA1 and 2) with similar size (**Figure 2C**). The 1.6 kbp-dsRNA segment was temporarily named dsRNA3 in the present research.

### Virus Particle

Virus particles were successfully purified from the mycelia of strain SCH941R6. Transmission electron microscope observation showed that the purified virus particles had a spherical morphology with a rough surface and 38 nm in diameter (**Figure 2A**). Three dsRNA segments released from purified virus particles were similar in size to those isolated directly from mycelia of strain SCH941R6 (**Figure 2B**). Furthermore, the yield of dsRNA3 from virus particles was lower than those of dsRNA1 and 2, which is consistent with that in mycelia (**Figure 2B**). Virus particles purified from the virus-infected Ep-1PNA367 strain were subjected to SDS-PAGE gel-electrophoresed analysis. The results showed that three major protein bands with approximate sizes of 120 kDa (p120), 100 kDa (p100), and 80 kDa (p80) were detected (**Figure 2D**, lane VP). These proteins were not detected in the preparations from the virus-free isogenic fungal strain following the same method as that used for the virus-infected strain (**Figure 2D**, lane VF). A protein with a size of 90 kDa (p90) was detected in both the virus-free and virus-infected preparations, and was thus likely a host protein co-fractionated with the virus particles (**Figure 2D**).

### Complete Sequencing of dsRNAs (1–3)

**FIGURE 3:**
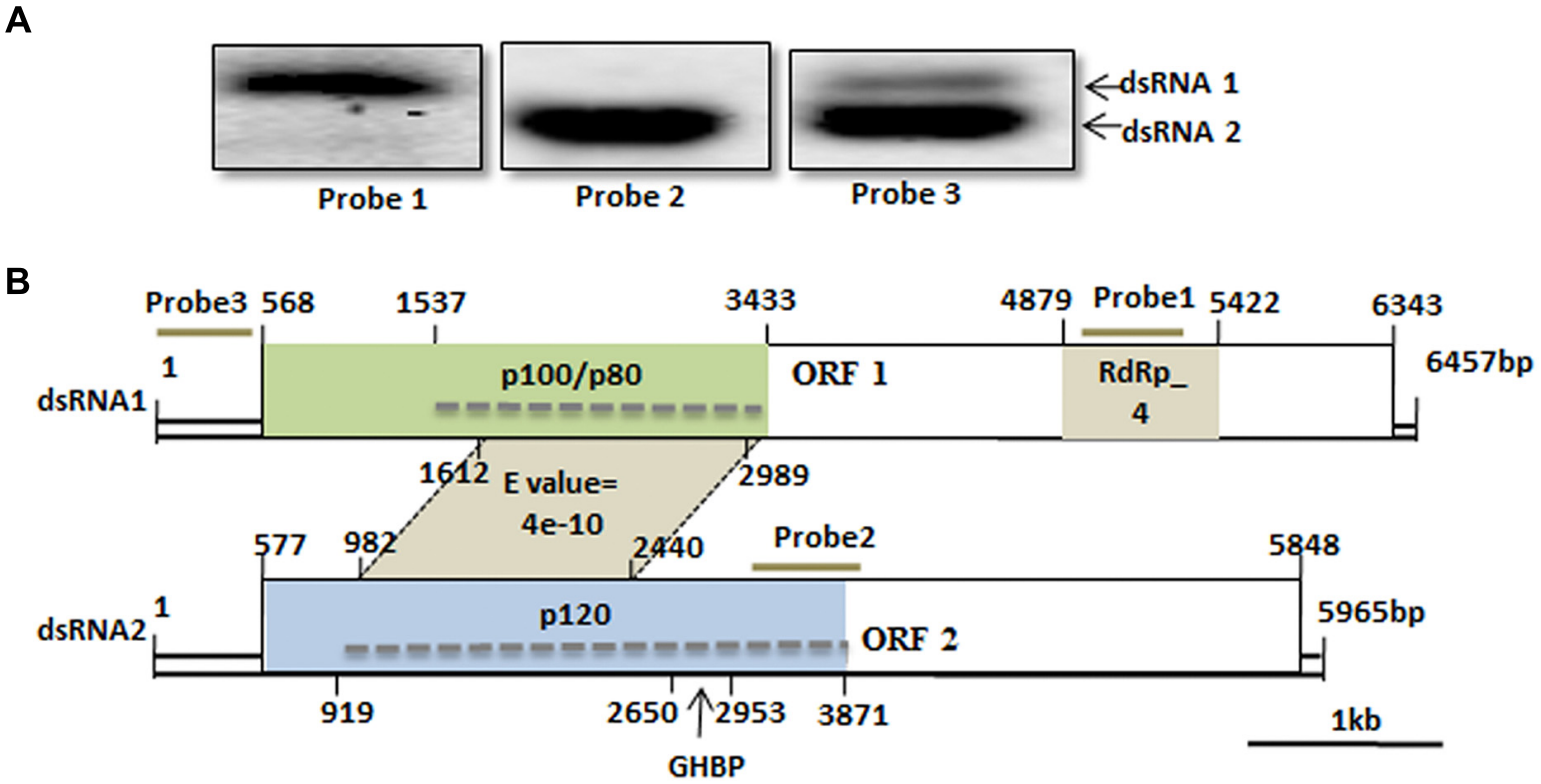
**Genome organization of SsBRV1. (A)** Northern blot analysis of SsBRV1 dsRNA1 and dsRNA2. dsRNA from viral particles were subjected to Northern blot analysis. The positions of probes 1 to 3 are shown in panel B. **(B)** Schematic representation of the genetic organization of dsRNA1 and dsRNA2 segments of the SsBRV1, and genome segment assignment of the structural proteins. dsRNA1 and 2 are 6,457 and 5,965 nt in length, respectively. dsRNA1 has a 568-nt-long 5′ UTR, one ORF (ORF1), and a 114-nt-long 3′ UTR, whereas dsRNA2 has a 577-nt-long 5′ UTR, one ORF(ORF2), and a 117-nt-long 3′ UTR. ORF1 and two are composed of 1,925 and 1,757 codons, respectively. Open boxes drawn with solid lines denote the ORFs. The gray position on ORF1 and 2 shows the conserved domain RdRp domain (RdRp_4, Pfam 02123). Polypeptides with sizes of approximately 120, 100, and 80 kDa corresponding to the region of the ORF1- and ORF2-encoded polyproteins are colored in light green and blue, respectively. The dashed line indicates the region of tryptic peptides identified by MS analysis (see Supplementary Table S3). The growth hormone receptor (GHR) binding domain (GHBP, Pfam 12772) in ORF2-encoded polyprotein is also indicated. The gray area between dsRNA1 and 2 indicates the conserved domain with significant similarity at protein level. The numbers above solid lines refer to the map positions of the initiation and termination codons of the respective ORF.

**FIGURE 4:**
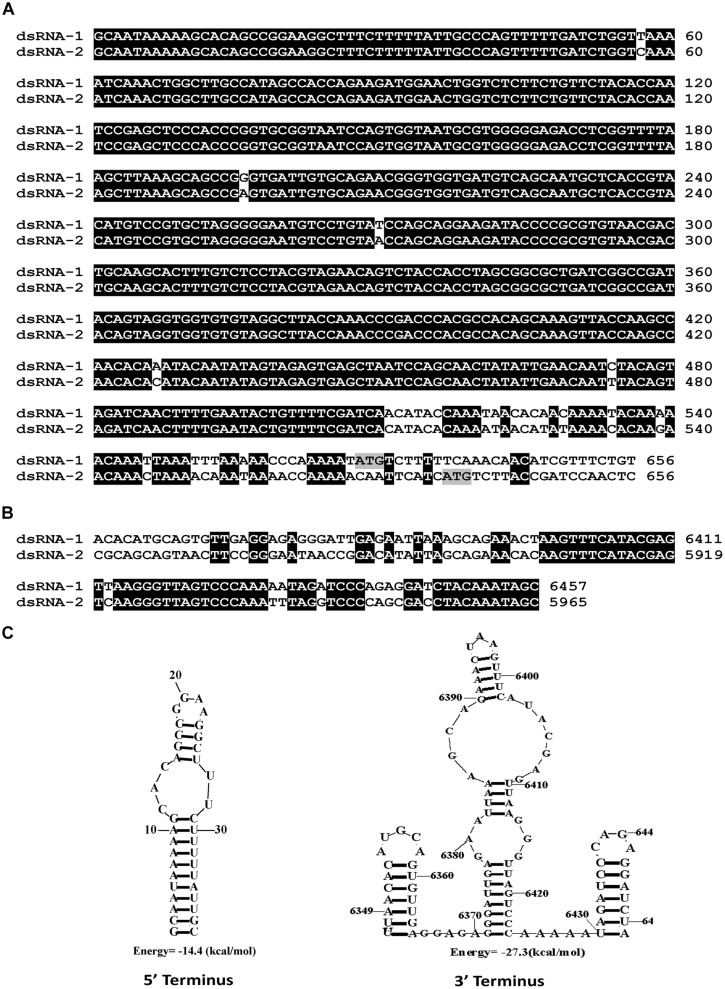
**Molecular characteristics of the SsBRV1, dsRNA1 and dsRNA2 segments. (A)** Conserved 5′ UTRs. The start codons of ORF1 and 2 are colored in gray. **(B)** Conserved 3′ UTRs. **(C)** Predicted secondary structures of the terminal sequences of the dsRNA1 and dsRNA2 segments.

We have cloned all the full-length cDNA sequences of dsRNAs (1–3) from strain SCH941R6. The full-length cDNA sequences for dsRNA1 (GenBank Acc. KP774592) and dsRNA2 (GenBank Acc. KP774593) of SsBRV1 were 6,457 and 5,965 bp (**Figure 3B**). The GC content of SsBRV1 was 49.5%. The 5′-untranslated region (UTR; 566 bp) and 3′-UTR (60 bp) of dsRNA1 and dsRNA2 are strictly conserved with 95 and 85% sequence identities, respectively (**Figures 4A,B**). Moreover, a Northern blot analysis was conducted to demonstrate the conservation of the 5′ UTRs (**Figure 3A**). This result also demonstrated that the yield of dsRNA1 from viral particles was much lower than that of dsRNA2 (**Figure 3A**). We also identified the possible secondary structures at the UTR and found one and three contiguous stem-loop structures were detected at the 5′ UTRs and 3′ UTRs, respectively (**Figure 4C**).

Only one large putative open reading frame (ORF1) was detected in dsRNA1 (nt 568-6343), and this ORF encodes a putative polyprotein of 1925 amino acids (aa’s) with a mass of approximate 217 kDa. This protein, named p1, contains a conserved RNA-dependent RNA polymerase (RdRp) domain (RdRp_4, Pfam02123; **Figure 3B**) at the C-terminal region, which has a low level of sequence similarity with the putative RdRps of some unclassified dsRNA viruses, i.e., *B. porri* RNA virus 1 (BpRV1), *Spissistilus festinus* virus 1 (SpFV1), *Circulifer tenellus* virus 1 (CiTV1), and members of *Totiviridae* and *Chrysoviridae* (Supplementary Table S1). These dsRNA viruses encompass monopartite, bipartite, or quadripartite dsRNA genomes. Among them, the highest bit score (875) and identity (38%) values were detected between the RdRp domains of SsBRV1 and the bipartite dsRNA virus BpRV1. The N-terminal region (aa position 14-891) of p1 also shares a significant sequence similarity with the corresponding region (aa position 29-920) of the BpRV1 ORF1-encoded polyprotein (Supplementary Table S2). However, no sequences in the NCBI database were detected to be homologous to the middle region (aa position 892-1436) in protein p1.

The dsRNA2 also has a single large ORF (ORF2; nt 577-5848), which encodes a large polyprotein with a mass of approximate 195 kDa. This polyprotein, named p2, shows a sequence similarity with the hypothetical protein encoded by the ORF2 of BpRV1. The regions (aa position 94-1009 and 1409-1730) of p2 were significantly similar to the corresponding regions (aa position 254-1148 and 1461-1761) of the BpRV1 ORF2-encoded polyprotein with 27 and 23% identifies, respectively (Supplementary Table S2). The other region of p2 (aa position 1010-1408) has no sequence similarity with any known sequence in the NCBI database.

**FIGURE 5:**
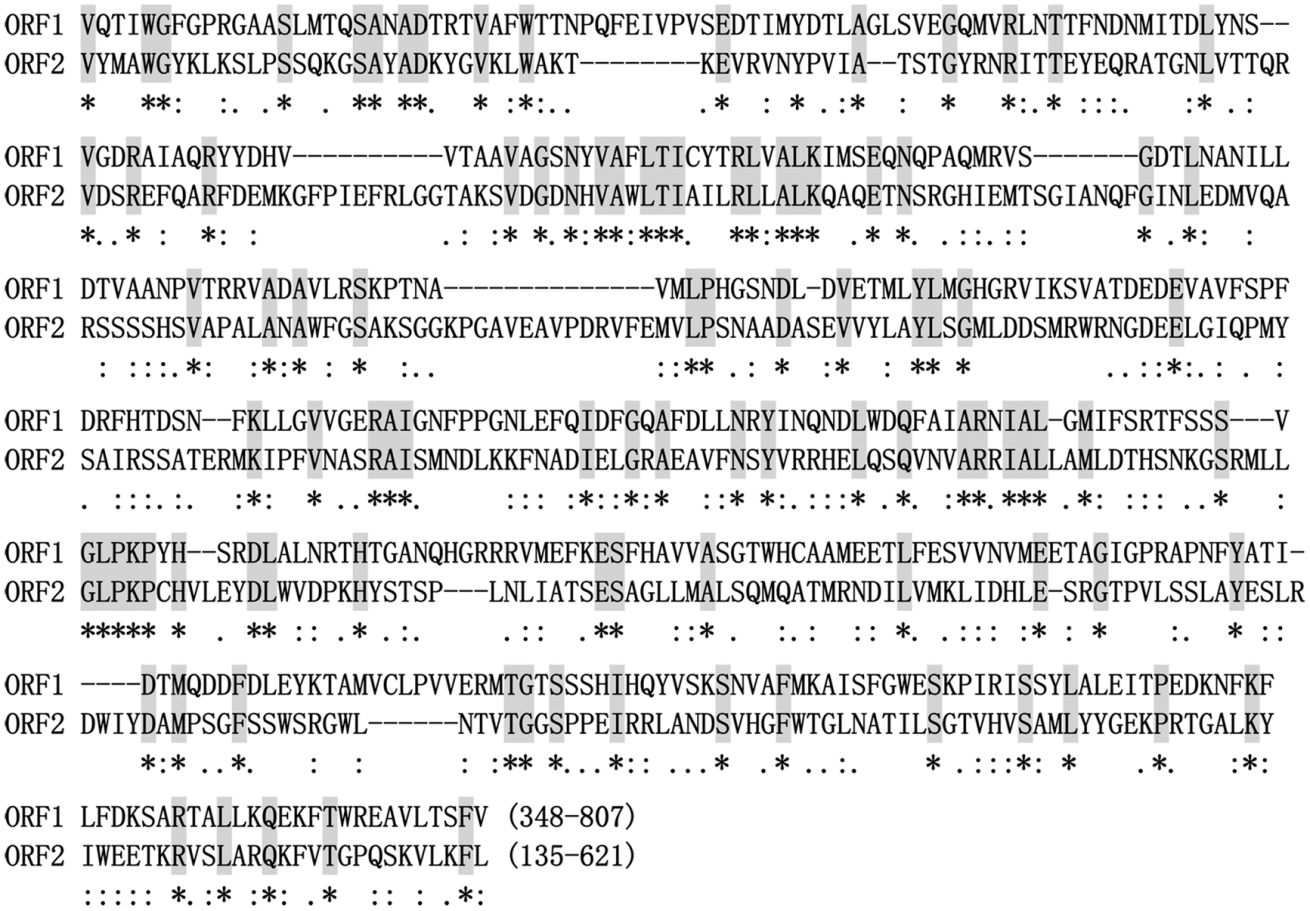
**Alignment of amino acid sequences with significant sequence similarity between SsBRV1 ORF1 and ORF2**. The identical amino acid residues are shaded in gray and indicated by asterisks. The conserved and semi-conserved amino acid residues are indicated by colons and dots.

Interestingly, a conserved domain that shows a sequence similarity with animal growth hormone receptor (GHR) binding domain (GHBP, Pfam12772, *E*-value = 3.88e-04) was detected in the p2 protein (aa position 691-792; **Figure 2B**). Multiple alignment analysis suggested that some conserved amino acid residues of the GHBP domain were identified in the p2 protein (Supplementary Figure S1). Moreover, a region between the polypeptide at position aa 348-807 of p1 and the polypeptide at position aa 135-621 of p2 was detected to have a significant sequence similarity (*E*-value = 4e-10; **Figure 3B**). Multiple sequence alignment also showed that there is an existence of widely conserved amino acid residues in those two regions (**Figure 5**). This property was also found for the bipartite dsRNA virus BpRV1 with a lower level of sequence similarity (*E*-value = 5e-06; Supplementary Figure S2). However, all of the typical bipartite dsRNA viruses, including members of *Birnaviridae*, *Picobirnaviridae,* and *Partitiviridae*, do not have a similar attribute between dsRNA1 and dsRNA2.

**FIGURE 6:**
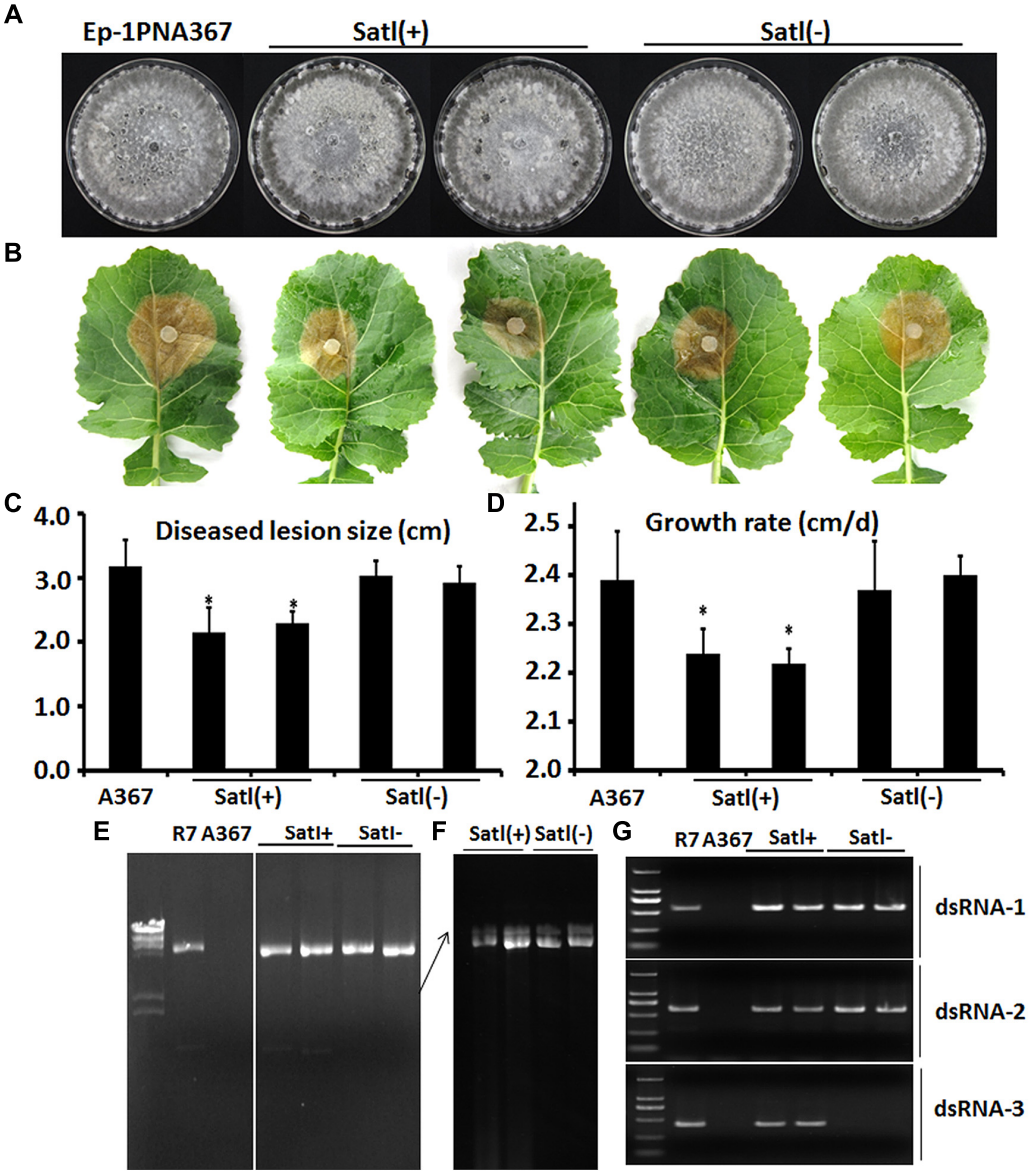
**Biological properties of SsBRV1-infected strains**. Satl(+) indicates SsBRV1-infected transfectants carrying the SatlRNA, whereas Satl(-) indicates SsBRV1-infected transfectants losing the SatlRNA. **(A)** Colony morphology. All of the strains were cultured on PDA for 10 days at 20–22∘C. **(B)** Virulence test on the detached leaves of rapeseed. **(C)** Statistical analysis of the lesion size. **(D)** Statistical analysis of the growth grate. The error bars indicate the SD from three sample means. “^∗^” are significantly different at the *P* < 0.05 level of confidence according to Duncan’s multiple range test. **(E)** 1% agarose gel-electrophoresed analysis of dsRNA from SsBRV1-infected transfectants. SCH941R7 (labeled with R7) was used as the positive control. **(F)** The upper dsRNA band from SsBRV1-infected transfectants was further isolated in 0.7% agarose gel and run in 0.5x TBE buffer for 16 h at 50 V. **(G)** RT-PCR confirmation of the SsBRV1-infected transfectants. SCH941R7 (labeled with R7) was used as the positive control. The predicted sizes of the RT-PCR products were 553, 611, and 487 nts.

The full-length cDNA of dsRNA3 is 1647 bp in length (GenBank Acc.KP774594), and its GC content is 46.9%. No major ORFs were found in dsRNA3, whereas one small ORF was deduced to encode a peptide with a mass of 13.7 kDa (121 aa; data not shown). However, sequence analysis revealed that no known proteins showed a significant similarity to dsRNA3 ORF-encoded proteins. Furthermore, the potential sequence relationship between dsRNA3 and dsRNA1 or dsRNA2 was not observed. The dsRNA3 can be lost during protoplasts transfection by SsBRV1 virions using PEG-mediated method (**Figures 6E–G**). Therefore, the dsRNA3 is not indispensable for virus SsBRV1, and we hypothesized it may be a satellite-like RNA (SatlRNA) and thus named it SatlRNA.

### Phylogenetic Analysis of SsBRV1

**FIGURE 7:**
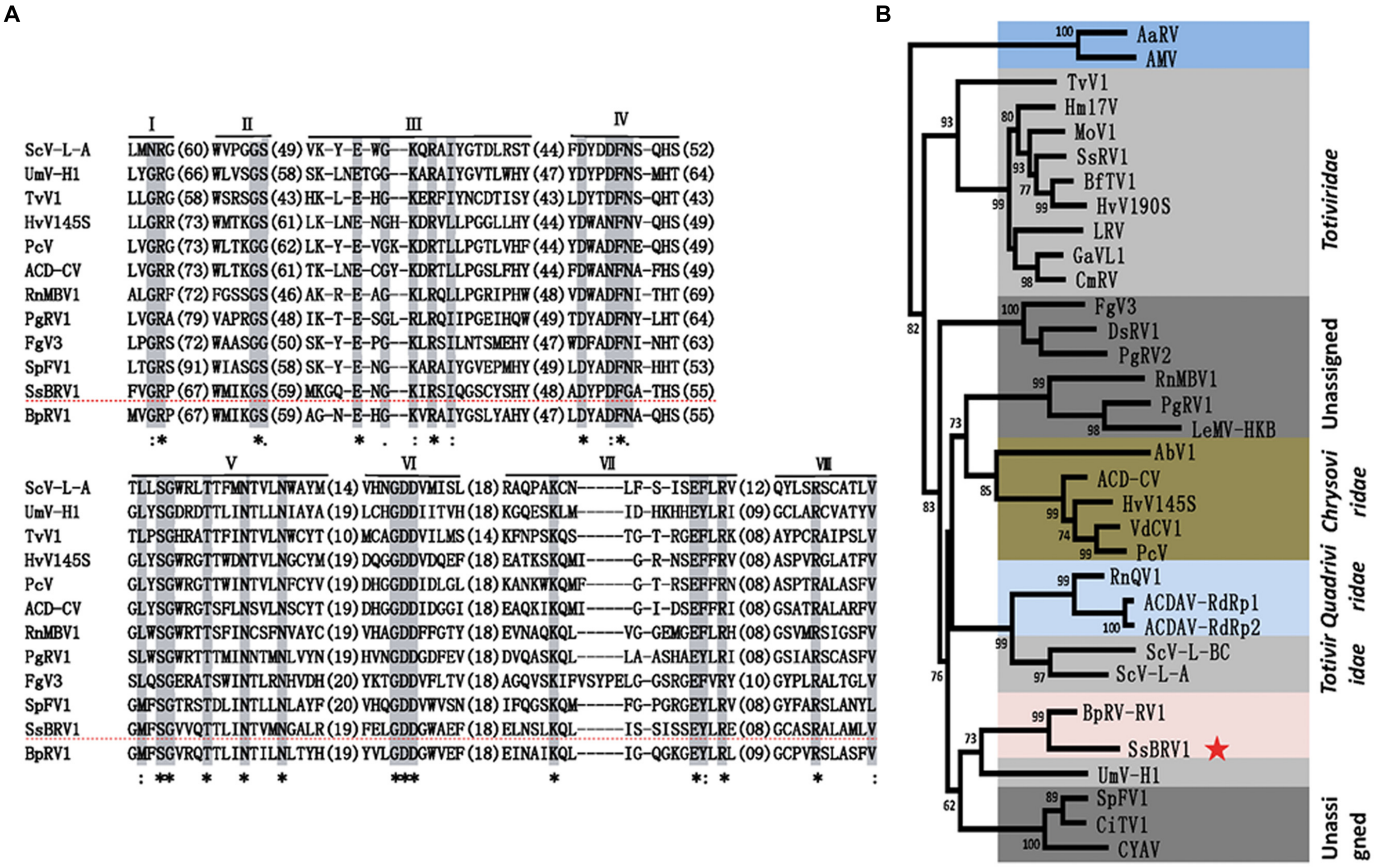
**Phylogenetic analysis of SsBRV1. (A)** Amino acid sequence alignment of the core RdRp motifs of SsBRV1 and selected viruses. The asterisks indicate identical amino acid residues, the colons represent highly chemically similar amino acid residues, and the dots indicate low chemically similar amino acid residues. Identical and chemically similar amino acid residues are shaded in gray and red. The dotted horizontal line shows SsBRV1. **(B)** Phylogenetic analysis of SsBRV1. Members of *Totiviridae*, *Chrysoviridae* and unassigned viruses related to SsBRV1 were selected to construct a maximum likelihood tree. The red star indicates the position of SsBRV1. Abbreviations of virus names and viral protein accession numbers used in the phylogenetic analysis were listed in Supplementary Table S4.

Multiple alignment analysis revealed that the RdRp domain of SsBRV1 contains eight conserved motifs in the C-terminus of the ORF1-encoded protein, which is widely present in dsRNA viruses (**Figure 7A**). A maximum-likelihood phylogenetic tree was constructed based on the RdRp domain. The phylogenetic tree indicated that SsBRV1 clusters with BpRV1 and a totivirus Ustilago maydis dsRNA virus-H1 (UmV-H1) to form a separated evolutionary clade (**Figure 7B**). BpRV1 is a bipartite dsRNA virus while UmV-H1 is a monopartite dsRNA virus (Kang et al., 2001; Wu et al., 2012). Clearly, SsBRV1 has a closest relationship with the bipartite dsRNA virus BpRV1 (**Figure 7B**, Supplementary Table S1). Therefore, SsBRV1 may be a new member of the proposed dsRNA virus family Botybirnaviridae. It is interested that SsBRV1 also shows close relationship to the monopartite virus UmV-H1 and the unassigned monopartite dsRNA viruses SpFV1 and CiTV1 that infect the insects three-cornered alfalfa hopper (*S. festinus*) and beet leafhopper (*C. tenellus*; **Figure 7B** and Supplementary Table S1; Spear et al., 2010). Based on the genome organization and characteristics, virus particles and phylogenic relationship, SsBRV1 ought to be a new member of the proposed virus family Botybirnaviridae and was named as *S.sclerotiorum* botybirnavirus 1 (SsBRV1).

### Analysis of Virus Structure Proteins

Three specific protein bands were identified in the preparations of SsBRV1 virus particle, i.e., p120, p100, and p80 (**Figure 2D**), and were individually cut and subjected to PMF-MS analysis in order to identify their corresponding genes. The results showed that p120, p100, and p80 generated a total of 45, 34, and 34 peptide fragments, respectively (Supplementary Table S3). Forty-five peptide fragments from p120 matched the ORF2-encoded polypeptide sequence at the aa position of 114 to 1098, accounting for 62.3% of the entire coverage (985 aa). Thirty-three peptide fragments from p100 matched the ORF1-encoded polypeptide at the aa position of 323 to 933, accounting for 90.8% of the entire coverage (611aa), whereas one peptide fragment from p100 matched the RdRp region of ORF1 at the aa position of 1842 to 1850 which may be degraded from the RdRp protein of SsBRV1. Thirty-four peptides from p80 also matched ORF1-encoded popypeptides at the aa position of 337 to 955, accounting for 87.6% of the entire coverage (619 aa). Based on the PMF-MS results, the genome segment assignment of the SsBRV1 structural proteins is presented in **Figure 3B**. Therefore, both ORF1 and 2 are involved in the structural proteins of SsBRV1 virus particles, and ORF1 actually encodes a cap-pol fusion protein.

### Biological Effects on *S. sclerotiorum*

Virus particles of SsBRV1 were purified from strain SCH941R6 and were used to successfully transfect the protoplasts of the virus-free strain Ep-1PNA367. SsBRV1 transfectants were confirmed by agarose gel-electrophoresed dsRNA isolation and RT-PCR detection (**Figures 6E–G**). These results suggested that the dsRNA3 segment was eliminated in some transfectants and may be a SatlRNA (**Figures 6E–G**). All of the SsBRV1-related strains were subjected to biological assessment. There was no significant difference in the colony morphology between the virus-free strain Ep-1PNA367 and the transfectants, regardless of the presence of SatlRNA (**Figure 6A**). However, in the presence of the SatlRNA, SsBRV1 can significantly reduce the virulence and growth rate of the transfectants (**Figures 6B–D**). There was no significant difference in the virulence and growth rate between the transfectants and the virus-free strain Ep-1PNA367 when the SatlRNA is absent (**Figures 6B–D**). Therefore, SsBRV1 carrying the SatlRNA can confer hypovirulence to the fungal host *S. sclerotiorum*.

## Discussion

In the present study, we isolated and characterized a novel bipartite dsRNA mycovirus SsBRV1 from the fungal plant pathogen *S. sclerotiorum*. SsBRV1 is phylogenetically related to BpRV1and ought to be a new member of the proposed virus family Botybirnaviridae, however, there are some unique properties that are distinct from BpRV1 and any other viruses.

### Biological Effect of SsBRV1 and Satellite RNA

There is a significant difference in the biological effects on the fungal hosts between SsBRV1 and BpRV1. SsBRV1 has no obvious effects on colony morphology (**Figure 6A**) and also has high pathogenicity on the detached leaves of rapeseed depending on the presence of the SatlRNA (**Figures 6B,C**). Nevertheless, BpRV1 leads to seriously debilitating symptoms in its fungal host *B. porri* (Wu et al., 2012). *S. sclerotiorum* and *B. porri* share many developmental and physiological features, and taxonomical relationship, and belong to the same taxonomic family *Sclerotiniaceae* (Amselem et al., 2011). It is interested that although SsBRV1 and BpRV1 may belong to the same virus family, they confer distinct biological effects to their fungal hosts.

The SatlRNA of SsBRV1 can significantly reduce the growth rate and virulence of strain Ep-1PNA367 (**Figures 6B–D**). Additional dsRNA segments (satellite or defective) may also be present in some bipartite dsRNA viruses (Rong et al., 2002; Tuomivirta and Hantula, 2005; Liu et al., 2008; Chiba et al., 2013; Zhang et al., 2013). Satellite RNAs are able to modulate, attenuate or exacerbate the symptoms caused by their cognate helper viruses (Kaper and Waterworth, 1977; Waterworth et al., 1979). The viral and satellite RNA conformations may play important roles in biological functions through regulating viral or host factors and employing replication signals and replicase complexes that are different from those used by their helper viruses (Annamalai et al., 2003; Huang et al., 2009; Simon and Gehrke, 2009). Moreover, defective dsRNA in a fungal partitivirus acts as an interfering RNA to influence the viral replication and the symptoms of the experimental fungus *C. parasitica* (Chiba et al., 2013). It appears that SsBRV1 SatlRNA may play some roles in modulating the virulence of SsBRV1 and further maintaining the balance between *S. sclerotiorum* and its hosts. However, the mechanism by which SsBRV1 SatlRNA affects the virulence of *S. sclerotiorum* remains largely unknown.

### Genome Organization and Characteristics

The dsRNA1 and dsRNA2 of SsBRV1 share a similar genetic organization, including a long 5′ UTR, one large ORF and a relatively short 3′ UTR. Although a conserved terminal property is frequently observed in multipartite viruses, this kind of long and strictly conserved 5′ UTR has not been observed in all of the known viruses with the exception of BpRV1 and RnMBV1. Notably, the first start codons (ATG) of ORF1 and ORF2 are not located in the strictly conserved 5′ UTR of SsBRV1 (**Figure 4A**), but occur in that of BpRV1 (Wu et al., 2012).

Although three structural proteins were identified in both SsBRV1 and BpRV1, there were also some significant differences among these proteins. In SsBRV1, the three structural proteins were p120, p100, and p80 with sizes of 120, 100, and 80 kDa (**Figure 2D**), while those of BpRV1 were p85, p80, and p70 with sizes of 85, 80, and 70 kDa, respectively (Wu et al., 2012). Moreover, SsBRV1 ORF2 was identified to encode the larger protein p120 and ORF1 was responsible for the smaller proteins p100 and p80. In BpRV1, the ORF1 encodes the larger protein p80 and p85 while ORF2 encodes the smaller protein p70 (Wu et al., 2012). We also note that the ratio of dsRNA1 and dsRNA2 is unequal in SsBRV1 but equal in BpRV1, and the yield of SsBRV1 dsRNA2 is more than that of dsRNA1 both in mycelia and virus particles (**Figure 2C**). Obviously, differences between SsBRV1 and BpRV1 in the molecular features, virus particles and biological effects may be dependent on the different fungal hosts.

We also identified a GHBP domain (Pfam 12772) in SsBRV1 ORF2-encoded protein, which was not detected in that of BpRV1 (**Figure 3B**). Growth hormone (GH) is a member of a large class of evolutionarily related protein hormones that have been cloned from human, mouse, rat, cow, chicken, pig, horse, and many other species (Kopchick and Andry, 2000). GH is known to be able to regulate the lipid, carbohydrate, nitrogen, and mineral metabolisms within a cell and exerts diverse and pleiotropic effects on cellular metabolism and differentiation (Kopchick and Andry, 2000). However, GH depends on its interaction with specific GHRs to promote its various effects. Growth hormone-binding proteins (GHBPs) are short forms of GHRs and are produced by proteolytical cleavage and/or alternative processing of the GHR transcript (Gonzalez et al., 2007). By competing with GHRs for binding GH, GHBPs may act as a reservoir or as negative forms of receptors to play an important role in balancing the physiological concentrations of GH (Gonzalez et al., 2007). It is hypothesized that there are some cytokines and similar pathways in fungi that resemble those of animals. Nonetheless, we did not find any homologs of GH and GHRs in any known fungal genomes. GHR proteins need to play some potential roles in signaling pathway except for binding the signaling molecules. In other words, a protein needs more than just the binding domain to be functional. As a lower eukaryote, fungi were distinct from animals in many ways. If the GHBP domain present in a protein plays a role in binding something, it’s likely unrelated to GHRs.

### The Possible Gene Expression Strantegy

Polypeptide mass fingerprint analysis showed the three structural proteins (p120, p100, p80) were encoded by ORF2 and ORF1, respectively. This finding prompts us a question that how these proteins are expressed. Because the peptides fragments from p100 include almost all of those from p80, p80 may be degraded from p100 or derived from p100 via a post-translational process resembling that of the related capsid proteins of the victorivirus *Helminthosporium victoriae* virus 190S (HvV190S; Huang and Ghabrial, 1996). Most monopartite dsRNA viruses contain two ORFs that are either overlapping or non-overlapping and linearly arranged in the genome responsible for the viral coat protein and RdRp protein (Wickner et al., 2011). UmV-H1 has been reported to have one large ORF encoding a polyprotein that contains structural motifs for capsid polypeptide, papain-like protease, and RdRp (Kang et al., 2001). In some birnaviruses, ORF1 also encodes a precursor protein (preVP2) that is auto-processed to generate mature capsid protein (VP2) during virus particle assembly (Delmas et al., 2011). It appears that both SsBRV1 ORFs are likely to use a similar expression strategy.

### Relationship with other dsRNA Viruses

There are obvious differences between SsBRV1 and the typical bipartite dsRNA viruses in genome size, the possible gene expression, structural protein components. In SsBRV1, there is a conserved region between ORF1 and ORF2 that shares significant similarity (**Figures 3B and 5**). Similarly, this attribute is also present in the corresponding region of BpRV1, indicating some conserved roles in these novel fungal bipartite dsRNA viruses (Supplementary Figures S2A,B). Typical bipartite viruses with two dsRNA genomes presently include birnaviruses, picobirnaviruses, and partitiviruses. However, SsBRV1 is distinct from these typical bisegmented dsRNA viruses. The complete genome of SsBRV1 is 12.4 kbp, larger than those of birnaviruses (5.9–6.9 kbp), picobirnaviruses (3.9–4.5 kbp) and partitiviruses (2.8–4.8 kbp; Supplementary Figure S3). The two dsRNA segments of bornaviruses and picobirnaviruses are encapsidated together, whereas dsRNA1 and dsRNA2 of partitiviruses are separately encapsidated (Delmas, 2011; Delmas et al., 2011; Ghabrial et al., 2011). Considering the genome size and the diameter of the virus particles, the two dsRNA segments of SsBRV1 is likely separately encapsidated. Moreover, the fact that the two dsRNA segments are present in unequal molar proportions (**Figures 2C and 6F**) further indicates the separate encapsidation of each dsRNA segment. Notably, smaller genome segment of birnaviruses and picobirnaviruses is responsible for viral replication, whereas larger genome segment encodes a polypeptide including the coat protein (Supplementary Figure S3; Delmas, 2011). However, in partitiviruses, larger genome segment encodes the viral RdRp, and smaller genome segment encodes the viral coat protein (Supplementary Figure S3; Ghabrial et al., 2011). Although SsBRV1, BpRV1 and these typical bipartite dsRNA viruses are composed of two dsRNA genome, there are obvious differences in genome size, gene expression and dsRNA encapsidation, indicating their diverse evolutional lineages.

However, SsBRV1 and BpRV1 have a close relationship with monopartite dsRNA viruses, i.e., members of *Totiviridae* and some unclassified dsRNA viruses, as well as the quadripartite dsRNA viruses quadriviruses and chrysoviruses (**Figure 7B**; Wu et al., 2012). There are also some similar molecular properties and virions attributes. These monopartite, bipartite, and quadripartite dsRNA viruses obviously share a closely evolutional relationship. The findings raise a question whether multipartite dsRNA viruses originated from monopartite dsRNA viruses or vice versa. Interestingly, RnMBV1 is a bipartite mycovirus with two large dsRNA segments, but its dsRNA2 can be lost in some isolates of *R. necatrix* (Kanematsu et al., 2014). Liu et al. (2012) noted that monopartite dsRNA viruses and even members of *Totiviridae* are not a monophyletic group and have diverse evolutionary lineages. It remains difficult to clarify the evolutionary history of these phylogenetically related monopartite and multipartite dsRNA viruses.

## Conclusion

We have characterized a novel bisegmented dsRNA virus SsBRV1 that is distinct from the typical bipartite dsRNA viruses, i.e., members of *Birnaviridae*, *Picobirnaviridae* and *Partitiviridae*, in terms of genome organization and molecular properties but was phylogenetically related to bipartite dsRNA virus BpRV1, monopartite dsRNA viruses and quadripartite dsRNA viruses. Three structural proteins were identified and involved in the formation of the virus particles. SsBRV1 virions can successfully transfect virus-free *S. sclerotiorum* strain and have limited impacts on the fungal hosts. A satellite-like dsRNA was closely related with the hypovirulence of SsBRV1-infected strains. SsBRV1 may be a new member of the virus family Botybirnaviridae.

## Conflict of Interest Statement

The authors declare that the research was conducted in the absence of any commercial or financial relationships that could be construed as a potential conflict of interest.

## Footnotes

1 http://blast.st-va.ncbi.nlm.nih.gov/Blast.cgi

2 http://tcoffee.crg.cat/apps/tcoffee/do:mcoffee

3 http://darwin.uvigo.es/software/prottest\_server.html

## References

[B1] Abascal F. Zardoya R. Posada D. (2005). ProtTest: selection of best-fit models of protein evolution. *Bioinformatics* 21 2104–2105 10.1093/bioinformatics/bti263 15647292

[B2] Amselem J. Cuomo C. A. Kan J. A. Viaud M. Benito E. P. Couloux A. (2011). Genomic analysis of the necrotrophic fungal pathogens *Sclerotinia sclerotiorum* and *Botrytis cinerea*. *PLoS Genet.* 7:e1002230 10.1371/journal.pgen.1002230 PMC3158057 21876677

[B3] Annamalai P. Hsu Y. H. Liu Y. P. Tsai C. H. Lin N. S. (2003). Structural and mutational analyses of cis-acting sequences in the 5′-untranslated region of satellite RNA of bamboo mosaic potexvirus. *Virology* 311 229–239 10.1016/S0042-6822(03)00178-8 12832220

[B4] Bolton M. D. Thomma B. P. H. J. Nelson B. D. (2006). *Sclerotinia sclerotiorum* (Lib.) de Bary: biology and molecular traits of a cosmopolitan pathogen. *Mol. Plant Pathol.* 7 1–16 10.1111/j.1364-3703.2005.00316.x 20507424

[B5] Chiba S. Lin Y. H. Kondo H. Kanematsu S. Suzuki N. (2013). Effects of defective interfering RNA on symptom induction by, and replication of, a novel partitivirus from a phytopathogenic fungus, *Rosellinia necatrix*. *J. Virol.* 87 2330–2234 10.1128/JVI.02835-12 23236074 PMC3571465

[B6] Chiba S. Salaipeth L. Lin Y. Sasaki A. Kanematsu S. Suzuki N. (2009). A novel bipartite double-stranded RNA mycovirus from the white root rot fungus *Rosellinia necatrix*: molecular and biological characterization, taxonomic considerations, and potential for biological control. *J. Virol.* 83 12801–12812 10.1128/JVI.01830-09 19828620 PMC2786845

[B7] Delmas B. (2011). “Family Picobirnaviridae,” in *Proceedings of the Ninth Report of the International Committee on Taxonomy of Viruses*, eds King A. M. Q. Elliot L. Adams M. J. Carstens E. B. (San Diego: Elsevier), 535–539.

[B8] Delmas B. Mundt E. Vakharia V. N. Wu J. L. (2011). “Family Birnaviridae,” in *Proceedings of the Ninth Report of the International Committee on Taxonomy of Viruses*, eds King A. M. Q. Elliot L. Adams M. J. Carstens E. B. (San Diego: Elsevier), 499–507.

[B9] Ghabrial S. A. Castón J. R. Jiang D. Nibert M. L. Suzuki N. (2015). 50-plus years of fungal viruses. *Virology* 10.1016/j.virol.2015.02.034 25771805

[B10] Ghabrial S. A. Nibert M. L. Maiss E. Lesker T. Baker T. S. Tao Y. J. (2011). “Family Partitiviridae,” in *Proceedings of the Ninth Report of the International Committee on Taxonomy of Viruses*, eds King A. M. Q. Elliot L. Adams M. J. Carstens E. B. (San Diego: Elsevier), 523–534.

[B11] Ghabrial S. A. Suzuki N. (2009). Viruses of plant pathogenic fungi. *Annu. Rev. Phytopathol.* 47 353–384 10.1146/annurev-phyto-080508-081932 19400634

[B12] Gonzalez L. Curto L. M. Miquet J. G. Bartke A. Turyn D. Sotelo A. I. (2007). Differential regulation of membrane associated-growth hormone binding protein (MA-GHBP) and growth hormone receptor (GHR) expression by growth hormone (GH) in mouse liver. *Growth Horm. IGF Res.* 17 104–112 10.1016/j.ghir.2006.12.002 17321774

[B13] Guindon S. Dufayard J. F. Lefort V. Anisimova M. Hordijk W. Gascuel O. (2010). New algorithms and methods to estimate maximum-likelihood phylogenies: assessing the performance of PhyML 3.0. *Syst. Biol.* 59 307–321 10.1093/sysbio/syq010 20525638

[B14] Hillman B. I. Supyani S. Kondo H. Suzuki N. (2004). A reovirus of the fungus *Cryphonectria parasitica* that is infectious as particles and related to the *Coltivirus* genus of animal pathogens. *J. Virol.* 78 892–898 10.1128/JVI.78.2.892-898.2004 14694120 PMC368758

[B15] Hu Z. Wu S. Cheng J. Fu Y. Jiang D. Xie J. (2014). Molecular characterization of two positive-strand RNA viruses co-infecting a hypovirulent strain of *Sclerotinia sclerotiorum*. *Virology* 464–465, 450–459 10.1016/j.virol.2014.07.007 25104554

[B16] Huang S. Ghabrial S. A. (1996). Organization and expression of the doublestranded RNA genome of *Helminthosporium victoriae* 190S virus, a totivirus infecting a plant pathogenic filamentous fungus. *Proc. Natl. Acad. Sci. U.S.A.* 93 12541–12546 10.1073/pnas.93.22.12541 8901618 PMC38028

[B17] Huang Y. W. Hu C. C. Lin C. A. Liu Y. P. Tsai C. H. Lin N. S. (2009). Structural and functional analyses of the 3′ untranslated region of bamboo mosaic virus satellite RNA. *Virology* 386 139–153 10.1016/j.virol.2009.01.019 19201437

[B18] Jiang D. Fu Y. Li G. Q. Ghabrial S. A. (2013). Viruses of the plant pathogenic fungus *Sclerotinia sclerotiorum*. *Adv. Virus Res.* 86 215–248 10.1016/B978-0-12-394315-6.00008-8 23498908

[B19] Kang J. G. Wu J. C. Bruenn J. A. Park C. M. (2001). The H1 double-stranded RNA genome of *Ustilago maydis* virus-H1 encodes a polyprotein that contains structural motifs for capsid polypeptide, papain-like protease, and RNA-dependent RNA polymerase. *Virus Res.* 76 183–189 10.1016/S0168-1702(01)00250-7 11410317

[B20] Kanematsu S. Shimizu T. Salaipeth L. Yaegashi H. Sasaki A. Ito T. (2014). Genome rearrangement of a mycovirus *Rosellinia necatrix* megabirnavirus 1 affecting its ability to attenuate virulence of the host fungus. *Virology* 450-451 308–315 10.1016/j.virol.2013.12.002 24503094

[B21] Kaper J. M. Waterworth H. E. (1977). Cucumber mosaic virus associated RNA 5: causal agent for tomato necrosis. *Science* 196 429–431 10.1126/science.196.4288.429 17776951

[B22] Khalifa M. E. Pearson M. N. (2013). Molecular characterization of three mitoviruses co-infecting a hypovirulent isolate of *Sclerotinia sclerotiorum* fungus. *Virology* 441 22–30 10.1016/j.virol.2013.03.002 23541082

[B23] Khalifa M. E. Pearson M. N. (2014). Characterisation of a novel hypovirus from *Sclerotinia sclerotiorum* potentially representing a new genus within the Hypoviridae. *Virology* 464–465, 441–449 10.1016/j.virol.2014.07.005 25108682

[B24] Kopchick J. J. Andry J. M. (2000). Growth hormone (GH) GH receptor and signal transduction. *Mol. Genet. Metab.* 71 293–314 10.1006/mgme.2000.3068 11001823

[B25] Li G. Wang D. Huang H. C. Zhou Q. (1996). Polymorphisms of *Sclerotinia sclerotiorum* isolated from eggplant in Jiamusi Heilongjiang Province. *Acta Phytopathologica Sin.* 26 237–242.

[B26] Lin Y. H. Chiba S. Tani A. Kondo H. Sasaki A. Kanematsu S. (2012). A novel quadripartite dsRNA virus isolated from a phytopathogenic filamentous fungus, *Rosellinia necatrix*. *Virology* 426 42–50 10.1016/j.virol.2012.01.013 22321722

[B27] Liu H. Fu Y. Jiang D. Li G. Xie J. Peng Y. (2009). A novel mycovirus that is related to the human pathogen hepatitis E virus and rubi-like viruses. *J. Virol*. 83 1981–1991 10.1128/JVI.01897-08 19073734 PMC2643757

[B28] Liu H. Fu Y. Xie J. Cheng J. Ghabrial S. A. Li G. (2012). Evolutionary genomics of mycovirus-related dsRNA viruses reveals cross-family horizontal gene transfer and evolution of diverse viral lineages. *BMC Evol. Biol.* 12:91 10.1186/1471-2148-12-91 PMC3483285 22716092

[B29] Liu L. Xie J. Cheng J. Fu Y. Li G. Yi X. (2014). Fungal negative-stranded RNA virus that is related to bornaviruses and nyaviruses. *Proc. Natl. Acad. Sci. U.S.A.* 111 12205–12210 10.1073/pnas.1401786111 25092337 PMC4143027

[B30] Liu W. Duns G. Chen J. (2008). Genomic characterization of a novel partitivirus infecting *Aspergillus ochraceus*. *Virus Genes* 37 322–327 10.1007/s11262-008-0265-6 18770020

[B31] Marzano S. Y. Hobbs H. A. Nelson B. D. Hartman G. L. Eastburn D. M. McCoppin N. K. (2015). Transfection of *Sclerotinia sclerotiorum* with in vitro transcripts of a naturally occurring interspecific recombinant of *Sclerotinia sclerotiorum* hypovirus 2 significantly reduces virulence of the fungus. *J. Virol.* 89 5060–5071 10.1128/JVI.03199-14 25694604 PMC4403457

[B32] Mathews D. H. Disney M. D. Childs J. L. Schroeder S. J. Zuker M. Turner D. H. (2004). Incorporating chemical modification constraints into a dynamic programming algorithm for prediction of RNA secondary structure. *Proc. Natl. Acad. Sci. U.S.A.* 101 7287–7292 10.1073/pnas.0401799101 15123812 PMC409911

[B33] Nuss D. L. (2005). Hypovirulence: mycoviruses at the fungal-plant interface. *Nat. Rev. Microbiol.* 3 632–642 10.1038/nrmicro1206 16064055

[B34] Osaki H. Wei C. Z. Arakawa M. Iwanami T. Nomura K. Matsumoto N. (2002). Nucleotide sequences of double stranded RNA segments from a hypovirulent strain of the white root rot fungus *Rosellinia necatrix*: possibility of the first member of the Reoviridae from a fungus. *Virus Gene* 25 101–107 10.1023/A:1020182427439 12206302

[B35] Potgieter A. C. Page N. A. Liebenberg J. Wright I. M. Landt O. Dijk A. A. (2009). Improved strategies for sequence-independent amplification and sequencing of viral dsRNA genomes. *J. Gen. Virol.* 90 1423–1432 10.1099/vir.0.009381-0 19264638

[B36] Rong R. Rao S. Scott S. W. Carner G. R. Tainter F. H. (2002). Complete sequence of the genome of two dsRNA viruses from *Discula destructiva*. *Virus Res.* 90 217–224 10.1016/S0168-1702(02)00178-8 12457976

[B37] Simon A. E. Gehrke L. (2009). RNA conformational changes in the life cycles of RNA viruses, viroids, and virus-associated RNAs. *Biochim. Biophys. Acta* 1789 571–583 10.1016/j.bbagrm.2009.05.005 19501200 PMC2784224

[B38] Spear A. Sisterson M. S. Yokomi R. Stenger D. C. (2010). Plant-feeding insects harbor double-stranded RNA viruses encoding a novel proline-alanine rich protein and a polymerase distantly related to that of fungal viruses. *Virology* 404 304–311 10.1016/j.virol.2010.05.015 20541786

[B39] Tamura K. Peterson D. Peterson N. Stecher G. Nei M. Kumar S. (2011). MEGA5: molecular evolutionary genetics analysis using maximum likelihood, evolutionary distance, and maximum parsimony methods. *Mol. Biol. Evol.* 28 2731–2739 10.1093/molbev/msr121 21546353 PMC3203626

[B40] Tuomivirta T. T. Hantula J. (2005). Three unrelated viruses occur in a single isolate of *Gremmeniella abietina var*. abietina type A. *Virus Res.* 110 31–39 10.1016/j.virusres.2004.12.005 15845253

[B41] Urayama S. Kato S. Suzuki Y. Aoki N. Le M. T. Arie T. (2010). Mycoviruses related to chrysovirus affect vegetative growth in the rice blast fungus *Magnaporthe oryzae*. *J. Gen. Virol.* 91 3085–3094 10.1099/vir.0.025411-0 20797967

[B42] Waterworth H. E. Kaper J. M. Tousignant M. E. (1979). CARNA 5, the small cucumber mosaic virus-dependent replicating RNA causal agent of lethal tomato necrosis, regulates disease expression. *Science* 204 845–847 10.1126/science.204.4395.845 17730528

[B43] Wickner R. B. Ghabrial S. A. Nibert M. L. Patterson J. L. Wang C. C. (2011). “Family Totiviridae,” in *Proceedings of the Ninth Report of the International Committee on Taxonomy of Viruses*, eds King A. M. Q. Elliot L. Adams M. J. Carstens E. B. (San Diego: Elsevier), 639–650.

[B44] Wu M. Jin F. Zhang J. Yang L. Jiang D. Li G. (2012). Characterization of a novel bipartite double-stranded RNA mycovirus conferring hypovirulence in the phytopathogenic fungus *Botrytis porri*. *J. Virol.* 86 6605–6619 10.1128/JVI.00292-12 22496220 PMC3393542

[B45] Xiao X. Cheng J. Tang J. Fu Y. Jiang D. Baker T. S. (2014). A novel partitivirus that confers hypovirulence on plant pathogenic fungi. *J. Virol.* 88 10120–10133 10.1128/JVI.01036-14 24965462 PMC4136314

[B46] Xie J. Ghabrial S. A. (2012). Molecular characterization of two mitoviruses co-infecting a hypovirulent isolate of the plant pathogenic fungus *Sclerotinia sclerotiorum*. *Virology* 428 77–85 10.1016/j.virol.2012.03.015 22520836

[B47] Xie J. Wei D. Jiang D. Fu Y. Li G. Ghabrial S. A. (2006). Characterization of debilitation-associated mycovirus infecting the plant-pathogenic fungus *Sclerotinia sclerotiorum*. *J. Gen. Virol.* 87 241–249 10.1099/vir.0.81522-0 16361437

[B48] Xie J. Xiao X. Fu Y. Liu H. Cheng J. Ghabrial S. A. (2011). A novel mycovirus closely related to hypoviruses that infects the plant pathogenic fungus *Sclerotinia sclerotiorum*. *Virology* 418 49–56 10.1016/j.virol.2011.07.008 21813149

[B49] Xu Z. Wu S. Liu L. Cheng J. Fu Y. Jiang D. (2015). A mitovirus related to plant mitochondrial gene confers hypovirulence on the phytopathogenic fungus *Sclerotinia sclerotiorum*. *Virus Res*. 197C 127–136 10.1016/j.virusres.2014.12.023 25550075

[B50] Yu X. Li B. Fu Y. Jiang D. Ghabrial S. A. Li G. (2010). A geminivirus-related DNA mycovirus that confers hypovirulence to a plant pathogenic fungus. *Proc. Natl. Acad. Sci. U.S.A.* 107 8387–8392 10.1073/pnas.0913535107 20404139 PMC2889581

[B51] Yu X. Li B. Fu Y. Xie J. Cheng J. Ghabrial S. A. (2013). Extracellular transmission of a DNA mycovirus and its use as a natural fungicide. *Proc. Natl. Acad. Sci. U.S.A.* 110 1452–1457 10.1073/pnas.1213755110 23297222 PMC3557086

[B52] Zhang R. Liu S. Chiba S. Kondo H. Kanematsu S. Suzuki N. (2014a). A novel single-stranded RNA virus isolated from a phytopathogenic filamentous fungus, *Rosellinia necatrix*, with similarity to hypo-like viruses. *Front. Microbiol*. 18:360 10.3389/fmicb.2014.00360 PMC4103508 25101066

[B53] Zhang T. Jiang Y. Dong W. (2014b). A novel monopartite dsRNA virus isolated from the phytopathogenic fungus *Ustilaginoidea virens* and ancestrally related to a mitochondria-associated dsRNA in the green alga *Bryopsis*. *Virology* 462-463 227–235 10.1016/j.virol.2014.06.003 24999047

[B54] Zhang T. Jiang Y. Huang J. Dong W. (2013). Genomic organization of a novel partitivirus from the phytopathogenic fungus *Ustilaginoidea virens*. *Arch. Virol.* 158 2415–2419 10.1007/s00705-013-1742-3 23732929

