# Supplementary material for: Molecular characterization of a bipartite double-stranded RNA virus and its satellite-like RNA co-infecting the phytopathogenic fungus Sclerotinia sclerotiorum
Source: Front Microbiol. 2015 May 6;6:406. doi: 10.3389/fmicb.2015.00406 (PMC4422086; doi:10.3389/fmicb.2015.00406)
Supplement: Supplementary file 1 [file Data_Sheet_1.PDF]

Supplemental Material

FIG S1 Amino acid sequence alignment of the GHBP domain among different species. The asterisks indicate identical amino acid residues, the colons represent the conserved amino acid residues, and the dots represent the semi-conserved amino acid residues. The selected species are listed as follows: CAVPO, *Caviaporcellus*(Q9JI97.1); SsBRV1; PAPAN, *Papioanubis* (Q9XSZ1.1); MOUSE, *Musmusculus* (Q3UP14); MONDO, *Monodelphisdomestica* (Q9N0Y7); COLLI, *Columba livia* (Q90375.1); PKLSJ, *Pelodiscussinensisjaponicus* (Q9DE35); ANGJA, *Anguilla japonica* (Q6L631); SASA, *Salmosalar*(NP_001135089.1); and CYSE, *Cynoglossussemilaevis* (ACM43288.1).

FIG S2 (A) Schematic representation of the polypeptide regions with significant similarity between BpRV1 ORF1 and ORF2. (B) Alignment of amino acid sequences with significant sequence similarity between BpRV1 ORF1 and ORF2. The identical amino acid residues are shaded in gray and indicated by asterisks. The conserved and semi-conserved amino acid residues are indicated by colons and dots.

FIG S3 Comparison of SsBRV1 and BpRV1 with the typical bipartite dsRNA viruses from *Birnaviridae*, *Picobirnaviridae*, and *Partitiviridae*. The selected viruses are listed as follows: BpRV1, Botrytis porri dsRNA virus 1; IPNV, Infectious pancreatic necrosis virus; IBDV, Infectious bursal disease virus; HPV, Human picobirnavirus; BCV1, Beet cryptic virus 1; and CcVF17, Cannabis cryptic virus isolate Fedora17.

Table S1. Summary of the results of a BLASTP search with ORF 1-encoded RdRp.

Table S2. Summary of the comparing results between the structural proteins between SsBRV1 and BpRV1.

Table S3. Peptide mass fingerprinting analysis of SsBRV1 structural proteins.

Table S4. Selected viruses used for phylogenetic analysis.

```
          . **  :   :* :   :                           :
CAVPO     GDSGRTSCYEPDILEADFNANDGTS---EDVQPDKL-KEEADL-LCLDEKNQNN---SPC-DAPPDPQQALVI-PPEEEKPQP--------------LLIGKTESTNQDA
SsBRV1    DASGLASHYEPAF---DFNN--------------AISFSSAVMNLGTKPKDGRHSSDPSTHDGNA-----------------P--------------VPMPRKRSNS--V
PAPAN     GDSGRTSCYEPDILETDFNANNIHEGTSEVAQPQRL-KGEADL-LCLDQKNQNK---SPYHDACPAAQQSSVI-QAEKNKPQP--------------LPTDGAESTHQAA
MOUSE     DDSGRTSCYDPDILDTDFHTSDMCDGTLKFAQSQKL-NMEADL-LCLDQKNLKN---LPY-DASLGSLHPSITQTVEENKPQP--------------LLSSETEATHQLA
MONDO     DDSGRTSCYEPDILETDLSAPASCNSTSDIAHSQRL-PGTADL-FCLEPKNEAS---PSTTPPPNGGL------LSEGGQPRP--------------LLPNGMESSSLAV
COLLI     DDSGRASCCEPDIPETDFSASDTCDAISDIDQFKKVTEKEEDL-LCLGRKDNDES-LPSLANTDTQQPRMSTR--PENSQPWP--------------PFADSIDAASPSA
PELSJ     DDSGRASCCEPDIPETDFSASDTCDGTSDIDQSKKTSEKEEDL-LCLDQKDNNES-HASPDDPTAQKPNTNIQ--SEDNQSKL--------------PFADSIESTRPPV
ANGJA     DDSGRASCYDPEIPDPEIP---------------ELPGPDPLA-LPKQGPGPAHL-TPPSSASP------------------S--------------PTT--VDRAHPLI
SASA      DDSGRARSYDPELPDQETL---------------MLMAA---L-LSSQPDKVEPC-LGNWSHSST-----------------PSIDVLEVPCPGLQAPGLPPEGRERHLV
CYSE      DDSGRASCYDPDLLDQDTPV--------------LVTT----L-LPGQPENGEAL-LDVVGGTST-----------------P-------------------ERSERPLV

                            *.:: *:* .      * .* :
CAVPO     PNQISNPISLANMDFYAQVSDITPAGSV------VLSPGQKNKAG-L-SQCEA-----------------------------H-------------------PE 382-515
SsBRV1    SAQLG-------------VDQISPAGRFFSEVFKVKQPKKKADSQEQPDKGDS-----------------------------K-------------------KL 691-793
PAPAN     HIQLSNPSSLANIDFYAQVSDITPAGSV------VLSPGQKNKAG-M-SQCDM-----------------------------H-------------------LE 382-519
MOUSE     STPMSNPTSLANIDFYAQVSDITPAGGV------VLSPGQKIKAG-I-AQGNT-----------------------------Q-------------------RE 393-530
MONDO     HAHLSNQSSLANIDFYAQVSDITPAGSV------VLSPGQKSKAG-I-SQCDG-----------------------------H-------------------RE 358-490
COLLI     HNQLSNQNSLRNTDFYAQVSDITPAGSV------VLSPGQKSKVA-R-ARCEF-----------------------------C-------------------TE 358-497
PELSJ     HTQLSNQSSGANTDFYAQVSNVTPAGSV------VLSPGQTNKRR-R-TQCEA-----------------------------Y-------------------TE 356-495
ANGJA     QTQLGT-QTWVNMDFYAQVSDVTPSGGV------VLSPGQQGGAPER-AEEEKKKKKEKKGEEEKGGEEREKEEEREKSKKKK-------------------KE 358-503
SASA      QTQLGGPQSWVNMDFYAQVSDVTPTGGV------VLSPGQQVRAPEN-TPTTE-----------------------------KDKKGKMEGGEEESEEVEERRM 378-518
CYSE      QIQTTGPQTWVNTDFYAQVSNVMPSGGV------VLSPGQQLRTQES-TSSTE-----------------------------Q-------------------EE 388-490
```

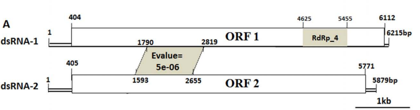

B

```
ORF1 GNNHIALGCTMYMRLMALEMLAEQGAATYIDLT---AEGFNRFLLPVEDGDDAPIRKVARSV-------VAHQPYNMITL
ORF2 GDNHIGMLVQWLIMLWSVRQAEEQGSTCHVRASSSAAEKYTLNVVPAKNTNETTEVKIMEAVAHAWEWAIKAKPFDLV-L
     *:***.:     : * ::.   ***:: ::  :   ** :.  ::*.:: :::.  *: .:*       :  :*:::  *

ORF1 PDQSADS-DAILMYYLAGNSRITTYVTAGPGAPDLAVHSTIDLYVPE----QRFTLC-PLNGSELIEERDSDDDGAFKVD
ORF2 PGDSANPIDIIHMMFLCGMLQDTTKFRYNK--TDLSIVPTYYTYQTDIREYLRIPLCNSLKALSLVDLSENE----YLMT
     *.:**:. * * * :*.*  : ** .  .   .**:: .*   * .:     *:.** .*:. .*::  :.:    : :

ORF1 AYRARSLVSRYLTAHNLWQQFPVMRAFAWALLAHPATSVNIQY----PAPMHTADLQLNLFPNPASIEHRGVILGERDHQ
ORF2 SGGLECIIIDYVRRMGLQDQLHNAQLIACSLIVSDISNSDSNLMGGIPKPNHVQEYELWL---STRAQGRGTTLTLMQGE
     :   ..::  *:   .* :* :   ::* :*:.   :. : :     * * *. : :* *   .:  : **. *   : :

ORF1 T--------SLHASV---VTAARMAEEVLTDSI----VSTVVEAGIEYTNPAYTGTVE--QELGRHLYHGYAHLIMPIVE
ORF2 SFALLFALLQIQRGVLIDVVAAKMIERVQSKGIPMISIAGEREAGIFLSEVLGSGASQLMSAWGSHMF-GY---VSAEQE
     :        .:: .*   *.**:* *.* :..*    ::   ****  ::   :*: :  .  * *:: **   : .  *

ORF1 KLLGDTFPELTQYLADSVLALEHCTTGRDLDRKKFRASSYLCMEETPEGEGWQVVFSESRR (462-805)
ORF2 KLLHTDAVMLTRYLEETF------TTGR------LRPTSCMLAGTTIETGAMSLIWDEATR (396-750)
     ***      **:** ::.      ****      :*.:* :    * *  . .:::.*: *
```

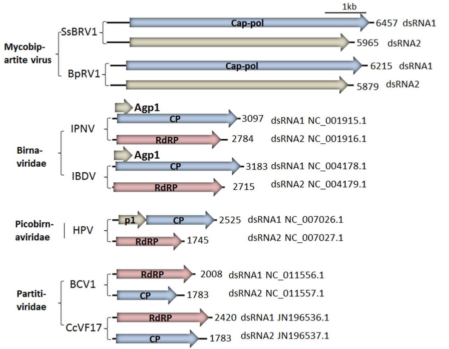
1kb
Mycobip-artite virus
SsBRV1
Cap-pol
6457 dsRNA1
5965 dsRNA2
BpRV1
Cap-pol
6215 dsRNA1
5879 dsRNA2
Birna-viridae
IPNV
Agp1
CP
3097 dsRNA1 NC_001915.1
RdRP
2784 dsRNA2 NC_001916.1
IBDV
Agp1
CP
3183 dsRNA1 NC_004178.1
RdRP
2715 dsRNA2 NC_004179.1
Picobirn-aviridae
HPV
p1
CP
2525 dsRNA1 NC_007026.1
RdRP
1745 dsRNA2 NC_007027.1
Partiti-viridae
BCV1
RdRP
2008 dsRNA1 NC_011556.1
CP
1783 dsRNA2 NC_011557.1
CcVF17
RdRP
2420 dsRNA1 JN196536.1
CP
1783 dsRNA2 JN196537.1

Table S1. Summary of the results of a BLASTP search with ORF 1-encoded RdRp.

Mycoviruses	RdRp size (aa)	RdRP_4 conserved region			BLAST			Accession NO.
		Conserved domain	Size (aa)	Identity %	Bit score	Coverage	E value	
**Unclassified**								
SsBRV1	1925	1437-1618	182					
BpRV1	1902	1347-1594	248	38	875	89%	7.00E-174	YP_006390636.1
PLV	1277	651-904	254	25	103	20%	1.00E-18	YP_009025166.1
SpFV1	1338	681-949	269	23	89.7	22%	2.00E-14	YP_003800001.1
CiTV1	1326	688-937	250	29	87.8	16%	8.00E-14	YP_003800003.1
RnMBV1	1111	508-761	254	23	67	13%	2.00E-07	YP_003288763.1
**Totiviridae**								
UmVH1	1820	1024-1530	507	25	119	25%	1.00E-23	NP_620728.1
UvRV1	823	196-571	376	31	86.7	12%	1.00E-13	YP_007761589.1
TcV2	1132	587-835	249	24	75.9	20%	3.00E-10	CBY84993.1
TvV2	1436	799-1229	431	29	71.2	14%	8.00E-09	AED99808.1
TvV3	1443	804-1235	432	29	67.8	12%	1.00E-07	AED99800.1
BbRV1	834	222-584	363	24	62.4	21%	4.00E-06	CCC42235.1
MoV1	832	109-575	467	22	61.2	23%	8.00E-06	YP_122352.1
PgRV1	1414	777-1061	285	23	60.8	21%	1.00E-05	YP_003541123.1
ScV-L-A	731	40-519	480	27	58.2	13%	7.00E-05	NP_620495.1
SsRV1	838	121-589	469	24	55.1	12%	5.00E-04	NP_047558.1
**Chrysovidae**								
PeCV1	1117	362-832	471	22	79	27%	3.00E-11	YP_392482.1
RsCV1	1138	575-853	279	23	79	27%	3.00E-11	AFE83590.1
ACDACV1	1087	541-806	288	23	72	26%	4.00E-09	CAH03664.1
CnCV1	962	539-822	284	21	69.7	25%	2.00E-08	ACT79255.1
GACV1	957	357-749	393	21	68.9	23%	3.00E-08	AD060926.1
AfCV1	1114	370-829	460	21	67.8	23%	8.00E-08	CAX48749.1
MoCV1	1127	571-831	261	21	67.4	26%	1.00E-07	YP_003858286.1
MoCV3	1127	557-831	275	21	63.5	26%	1.00E-06	YP_008914864.1
VdCV1	1108	554-823	270	22	59.3	24%	3.00E-05	ADG21213.1
**Potyviridae**								
CYAV	634	26-311	286	23	58.2	16%	6.00E-05	CAA63099.2
**Partitiviridae**								
FgRV2	1137	572-846	275	22	61.2	22%	8.00E-06	ADW08802.1

Table S2. Summary of the comparing results between the structural proteins between SsBRV1 and BpRV1.

Genes	Virus name	Size(aa)	Coverage	Identity	Evalue	Bit score
ORF1	SsBRV1	1925	14-891	26%	1e-73	284
	BpRV1	1902	29-920			
ORF2	SsBRV1	1757	94-1009	27%	4e-94	348
	BpRV1	1788	254-1148			
ORF2	SsBRV1	1757	1409-1730	23%	4e-09	72.4
	BpRV1	1788	1461-1761			

Table S3. Peptide mass fingerprinting analysis of SsBRV1 structural proteins.

Peptide mass fingerprinting analysis of p120.

Amino acid sequence	Observed Mass	Calculated Mass	± delta	Ions score	Frequency	Position	Putative ORF
ELTLHAK	810.4641	810.4599	0.0042	24.47	2	886 - 892	ORF Ⅱ
GTLIQPAK	826.4965	826.4912	0.0053	37.49	2	804 - 811	ORF Ⅱ
SLAANLSR	830.4681	830.461	0.0071	49.94	1	1060 - 1067	ORF Ⅱ
MPDINFK	863.4272	863.4211	0.0061	43.66	2	114 - 120	ORF Ⅱ
GFPIEFR	864.4551	864.4494	0.0057	34.55	3	219 - 225	ORF Ⅱ
YIWEETK	967.4723	967.4651	0.0072	28.05	1	594 - 600	ORF Ⅱ
KIDSSLGVR	973.561	973.5556	0.0054	52.36	3	651 - 659	ORF Ⅱ
LIDHLESR	981.531	981.5243	0.0067	56.99	3	500 - 507	ORF Ⅱ
AISMNDLKK	1018.555	1018.548	0.0071	29.63	1	385 - 393	ORF Ⅱ
FNGLAETLR	1019.548	1019.54	0.0075	47.88	1	815 - 823	ORF Ⅱ
FNADIELGR	1033.527	1033.519	0.0077	43.81	3	393 - 402	ORF Ⅱ
ITTEYEQR	1038.506	1038.498	0.0075	45.35	2	187 - 194	ORF Ⅱ
ATGNLVTTQR	1059.573	1059.567	0.0055	47.4	3	195 - 204	ORF Ⅱ
STNTVTGEPR	1060.522	1060.515	0.0074	53.14	1	660 - 669	ORF Ⅱ
YIWEETKR	1123.574	1123.566	0.0074	46.02	2	594 - 601	ORF Ⅱ
LFDQGGVGSVR	1133.59	1133.583	0.0072	56.89	2	793 - 803	ORF Ⅱ
AEAVFNSYVR	1154.579	1154.572	0.0071	46.27	4	403 - 412	ORF Ⅱ
KFNADIELGR	1161.622	1161.614	0.0076	8.97	6	393 - 402	ORF Ⅱ
NPFLVQAPTAK	1184.664	1184.655	0.0089	55.12	1	121 - 131	ORF Ⅱ
KLFDQGGVGSVR	1261.687	1261.678	0.0092	60.33	2	792 - 803	ORF Ⅱ

HELQSQVNVAR	1279.667	1279.663	0.0038	13.55	4	414 - 424	ORF Ⅱ
IALLAMLDTHSNK	1425.772	1425.765	0.0075	37.69	5	425 - 438	ORF Ⅱ
RHELQSQVNVAR	1435.772	1435.764	0.0072	12.44	13	413 - 424	ORF Ⅱ
VNYPVIATSTGYR	1439.751	1439.741	0.0103	67.47	3	172 - 184	ORF Ⅱ
IALLAMLDTHSNK	1441.767	1441.76	0.0072	80.65	3	425 - 438	ORF Ⅱ
GTPVLSSLAYESLR	1491.804	1491.793	0.0109	76.25	2	508 - 521	ORF Ⅱ
FVRDEQWSGTVSK	1537.735	1537.753	-0.0173	0.32	1	1229 - 1241	ORF Ⅱ
AQDIEYTYAPLGAK	1538.772	1538.762	0.0105	78.47	2	869 - 882	ORF Ⅱ
RIALLAMLDTHSNK	1581.876	1581.866	0.0101	23.79	2	425 - 438	ORF Ⅱ
GWLNTVTGGSPPEIR	1582.822	1582.81	0.0111	97.68	3	538 - 552	ORF Ⅱ
LDDIPEQLKNPEYAK	1771.912	1771.899	0.0123	67.83	4	833 - 847	ORF Ⅱ
SNSVSAQLGVDQISPAGR	1784.915	1784.902	0.0137	57.25	2	747 - 764	ORF Ⅱ
NGDEELGIQPMYSAIR	1791.86	1791.846	0.0143	39.92	2	353 - 368	ORF Ⅱ
SVTYDESFPALPTAAEK	1824.901	1824.878	0.0225	39.17	1	1082 - 1098	ORF Ⅱ
SVDGDNHVAWLTIAILR	1878.996	1878.995	0.0003	15.34	5	232 - 248	ORF Ⅱ
HSSDPSTHDGNAPVPMPR	1900.857	1900.849	0.0086	42.49	7	727 - 744	ORF Ⅱ
DWIYDAMPSGFSSWSR	1903.829	1903.82	0.0095	27.75	2	522 - 537	ORF Ⅱ
HSSDPSTHDGNAPVPMPR	1916.852	1916.844	0.0085	21.12	4	727 - 744	ORF Ⅱ
LIYLGLRPAIVQYEER	1932.094	1932.083	0.0112	20.16	5	670 - 685	ORF Ⅱ
SQVLFVHLQDSHYEAYK	2063.035	2063.011	0.0235	9.39	1	990 - 1006	ORF Ⅱ
ANIFDTNPTDWMHYLADFSK	2385.083	2385.074	0.0093	114.26	4	626 - 645	ORF Ⅱ
ALQTASVVNNIKPYLELNALFK	2445.38	2445.363	0.0173	7	1	906 - 927	ORF Ⅱ
GHIEMTSGIANQFGINLEDMVQAR	2646.268	2646.253	0.0147	98.01	1	262 - 285	ORF Ⅱ
VFEMVLPSNAADASEVVYLAYLSGMLDDSMR	3408.618	3408.593	0.0256	3.11	1	320 - 350	ORF Ⅱ
MSEAQDASGLASHYEPAFDFNNAISFSSAVMNLGTKPK	4031.886	4031.867	0.019	12.25	3	686 - 723	ORF Ⅱ

Peptide mass fingerprinting analysis of p100.

Amino acid sequence	Observed Mass	Calculated Mass	±delta	Ions score	Frequency	Position	Putative ORF
SNVAFMK	795.3996	795.3949	0.0047	49.92	1	744 - 750	ORF Ⅰ
SLTRVTR	831.4874	831.4926	-0.0052	12.68	2	873 - 879	ORF Ⅰ
LLGVVGER	841.5078	841.5022	0.0057	53.17	2	564 - 571	ORF Ⅰ
FELHEPK	898.4595	898.4548	0.0047	13.81	4	934 - 940	ORF Ⅰ
RVADAVLR	898.5395	898.5348	0.0047	31.76	2	499 - 506	ORF Ⅰ
FHTDSNFK	994.4563	994.4509	0.0054	10.75	6	556 - 563	ORF Ⅰ
THTGANQHGR	1077.512	1077.506	0.0058	16.45	2	643 - 652	ORF Ⅰ
SIPEPEQQR	1082.541	1082.536	0.0049	10.72	4	947 - 955	ORF Ⅰ
NIALGMIFSR	1120.612	1120.606	0.0054	23.2	37	612 - 621	ORF Ⅰ
TAMVCLPVVER	1273.66	1273.652	0.0079	39.72	9	718 - 728	ORF Ⅰ
NISPQFEELER	1360.668	1360.662	0.0059	33.7	2	323 - 333	ORF Ⅰ
AISFGWESKPIR	1389.745	1389.741	0.004	29.13	10	751 - 762	ORF Ⅰ
IMSEQNQPAQMR	1431.668	1431.66	0.0077	26.9	14	465 - 476	ORF Ⅰ
GAASLMTQSANADTR	1492.703	1492.694	0.0086	79.1	7	358 - 372	ORF Ⅰ
ISSYLALEITPEDK	1577.826	1577.819	0.007	51.22	43	763 - 776	ORF Ⅰ
MTGTSSSHIHQYVSK	1661.79	1661.783	0.0065	95.29	10	729 - 743	ORF Ⅰ
TFSSSVGLPKPYHSR	1661.862	1661.853	0.0097	2.62	8	622 - 636	ORF Ⅰ
MTGTSSSHIHQYVSK	1677.786	1677.778	0.0078	57.55	7	729 - 743	ORF Ⅰ
FYGEFYDDNISDLR	1752.773	1752.763	0.0096	94.86	2	820 - 833	ORF Ⅰ
YINQNDLWDQFAIAR	1865.92	1865.906	0.0136	102.2	3	597 - 611	ORF Ⅰ
SVATDEDEVAVFSPFDR	1882.867	1882.859	0.0085	89.95	6	539 - 555	ORF Ⅰ
ISSYLALEITPEDKNFK	1967.04	1967.025	0.0144	13.6	1	763 - 779	ORF Ⅰ
DLNEEEAMQVDSLWATMK	2108.952	2108.939	0.0129	48.85	2	880 - 897	ORF Ⅰ

VTSGSGVQSYLVQTIWGFGPR	2238.159	2238.143	0.0154	90.33	1	337 - 357	ORF Ⅰ
VSGDTLNANILLDTVAANPVTR	2253.21	2253.197	0.0133	88.02	3	477 - 498	ORF Ⅰ
LNTTFNDNMITDLYNSVGDR	2302.07	2302.054	0.0159	101.1	5	410 - 429	ORF Ⅰ
EAVLTSFVCHSQWGPSEQHR	2354.093	2354.086	0.0063	14.84	8	800 - 819	ORF Ⅰ
APNFYATIDTMQDDFDLEYK	2396.062	2396.052	0.0103	16.57	8	698 - 717	ORF Ⅰ
SQLEHLEGSPTIWNTSTAATPLEFTKPK	3082.577	3082.561	0.0161	22.33	6	845 - 872	ORF Ⅰ
SKPTNAVMLPHGSNDLDVETMLYLMGHGR	3182.552	3182.531	0.0213	23.18	5	507 - 535	ORF Ⅰ
AALEAASAENDAEEDEDDEEFEYPQPTANPTGQGQR	3893.637	3893.626	0.0112	9.36	1	898 - 933	ORF Ⅰ
TVAFWTTNPQFEIVPVSEDTIMYDTLAGLSVEGQMVR	4144.048	4144.017	0.0302	80.98	2	373 - 409	ORF Ⅰ
ESFHAVVASGTWHCAAMEETLFESVVNVMEETAGIGP	4163.941	4163.903	0.0384	45.28	3	660 - 697	ORF Ⅰ
YMTEESITR	1128.52	1128.512	0.0083	0.67	1	1842 - 1850	ORF Ⅰ

Peptide mass fingerprinting analysis of p80.

**Amino acid sequence**	**Observed Mass**	**Calculated Mass**	**±delta**	**Ions score**	**Frequency**	**Position**	**Putative ORF**
DLALNR	700.3905	700.3868	0.0038	20.96	1	637 - 642	ORF Ⅰ
VADAVLR	742.4377	742.4337	0.004	31.9	2	499 - 506	ORF Ⅰ
SNVAFMK	795.4004	795.3949	0.0055	42.98	1	744 - 750	ORF Ⅰ
RVMEFK	808.4317	808.4265	0.0051	17.77	3	654 - 659	ORF Ⅰ
LLGVVGER	841.5074	841.5022	0.0053	55.49	3	564 - 571	ORF Ⅰ
RVADAVLR	898.5395	898.5348	0.0047	38.99	3	499 - 506	ORF Ⅰ
TALLKQEK	929.5588	929.5545	0.0043	16.24	2	788 - 795	ORF Ⅰ
FHTDSNFK	994.4566	994.4509	0.0057	32.78	7	556 - 563	ORF Ⅰ
THTGANQHGR	1077.512	1077.506	0.0054	29.38	5	643 - 652	ORF Ⅰ
SIPEPEQQR	1082.541	1082.536	0.0049	9.23	1	947 - 955	ORF Ⅰ

NIALGMIFSR	1120.614	1120.606	0.0081	52.68	63	612 - 621	ORF I
TAMVCLPVVER	1273.657	1273.652	0.0044	33.66	11	718 - 728	ORF I
NISPQFEELER	1360.672	1360.662	0.0095	72.88	4	323 - 333	ORF I
AISFGWESKPIR	1389.747	1389.741	0.0062	58.49	18	751 - 762	ORF I
IMSEQNQPAQMR	1431.667	1431.66	0.0076	88.71	21	465 - 476	ORF I
GAASLMTQSANADTR	1492.703	1492.694	0.0089	90.45	24	358 - 372	ORF I
ISSYLALEITPEDK	1577.822	1577.819	0.0031	77.48	67	763 - 776	ORF I
MTGTSSSHIHQYVSK	1661.792	1661.783	0.0086	83.63	15	729 - 743	ORF I
TFSSSVGLPKPYHSR	1661.862	1661.853	0.0092	51.03	11	622 - 636	ORF I
MTGTSSSHIHQYVSK	1677.788	1677.778	0.0096	107.25	15	729 - 743	ORF I
FYGEFYDDNISDLR	1752.77	1752.763	0.0067	57.88	10	820 - 833	ORF I
YINQNDLWDQFAIAR	1865.918	1865.906	0.0118	101.64	6	597 - 611	ORF I
SVATDEDEVAVFSPFDR	1882.857	1882.859	-0.0011	89.37	11	539 - 555	ORF I
ISSYLALEITPEDKNFK	1967.032	1967.025	0.0073	23.45	7	763 - 779	ORF I
VTSGSGVQSYLVQTIWGFGPR	2238.155	2238.143	0.0113	102.13	2	337 - 357	ORF I
VSGDTLNANILLDTVAANPVTR	2253.211	2253.197	0.0143	127.94	7	477 - 498	ORF I
LNTTFNDNMITDLYNSVGDR	2302.067	2302.054	0.013	59.26	10	410 - 429	ORF I
EAVLTSFVCHSQWGPSEQHR	2354.097	2354.086	0.011	59.57	11	800 - 819	ORF I
APNFYATIDTMQDDFDLEYK	2396.06	2396.052	0.0086	73.28	16	698 - 717	ORF I
SQLEHLEGSPTIWNTSTAATPLEFTKPK	3082.582	3082.561	0.0209	79.2	6	845 - 872	ORF I
SKPTNAVMLPHGSNDLDVETMLYLMGHGR	3182.547	3182.531	0.016	53.7	17	507 - 535	ORF I
TVAFWTTNPQFEIVPVSEDTIMYDTLAGLSVEGQMVR	4144.044	4144.017	0.0269	71.78	5	373 - 409	ORF I
ESFHAVVASGTWHCAAMEETLFESVVNVMEETAGIGPR	4163.926	4163.903	0.0228	33.69	2	660 - 697	ORF I
TVAFWTTNPQFEIVPVSEDTIMYDTLAGLSVEGQMVR	4176.045	4176.007	0.0374	50.91	1	373 - 409	ORF I

Table S4. Selected viruses used for phylogenetic analysis.

Virus Family	Virus Name	Abbreviation	Genbank accession No.
**Totividae**	Ustilago maydis virus H1	UmVH1	NP_620728.1
	Saccharomyces cerevisiae virus L-A	ScV-L-A	AAA50508.1
	Saccharomyces cerevisiae virus La	ScV-L-BC	AAB02146.1
	Helicobasidium mompa No.17 dsRNA virus	Hm17V	BAC81754.1
	Sphaeropsis sapinea RNA virus 1	SsRV1	NP_047558.1
	Magnaporthe oryzae virus 1	MoV1	BAD60833.1
	Gremmeniella abietina RNA virus	GaVL1	AAK11656.1
	Botryotinia fuckeliana totivirus 1	BfTV1	CAM33265.1
	Trichomonas vaginalis virus 1	TVV1	AET81012.1
	Helminthosporium victoriae virus	HvV190S	AAB94791.2
	Leishmania RNA virus 1 - 1	LRV	NP_041191.1
	Gremmeniella abietina RNA virus L1	GaVL1	AAK11656.1
	Coniothyrium minitans RNA virus	CmRV	YP_392467.1
	Zygosaccharomyces bailii virus Z	ZbV-Z	NP_624325.1
**Chrysoviridae**	Amasya cherry disease associated chrysovirus	ACD-CV	YP_001531163.1
	Verticillium dahliae chrysovirus 1	VdCV	ADG21213.1
	Agaricus bisporus virus 1	AbV1	CAA64144.1
	Helminthosporium victoriae 145S virus	HvV145S	AAM68953.1
	Penicillium chrysogenum virus	PcV	YP_392482.1
**Quadriviridae**	Rosellinia necatrix quadrivirus 1	RnQV1	BAL46425.1
**Partitiviridae**	Amasya cherry disease-associated mycovirus	ACDAV-RdRp1	CAJ29958.1

	RNA-dependent RNA polymerase 1<br>Amasya cherry disease-associated mycovirus<br>RNA-dependent RNA polymerase 2	ACDAV-RdRp2	CAJ29959.1
**Unassigned**	Spissistilus festinus virus 1	SpFV1	ADK12922.1
	Circulifer tenellus virus 1	CiTV1	ADK12924.1
	Cucurbit yellows-associated virus	CYAV	CAA63099.2
	Phlebiopsis gigantea mycovirus dsRNA 1	PgRV1	CAJ34333.2
	Fusarium graminearum dsRNA mycovirus-3	FgV3	YP_003288789.1
	Diplodia scrobiculata RNA virus 1	DsRV1	YP_003359178.1
	Phlebiopsis gigantea mycovirus	PgRV2	CAJ34335.2
	Lentinula edodes mycovirus HKB	LeV-HKB	BAG71788.2
	Rosellinia necatrix megabirnavirus 1/W779	RnBMV1	BAI48016.1
	Botrytis porri dsRNA virus 1	BpRV1	YP_006390636.1
	Alternaria alternata virus 1	AaRV	YP_001976142.1
	Aspergillus mycovirus 341	AMV	ABX79997.1
